# Mitotic Cdc42 waves encode PI(3,4)P_2_ signaling and Golgi morphological state to control spindle scaling

**DOI:** 10.1126/sciadv.aec7705

**Published:** 2026-06-19

**Authors:** Suet Yin Sarah Fung, Shengping Xiao, Yujin Bao, Morven Graham, Maohan Su, Xinran Liu, Joerg Bewersdorf, Min Wu

**Affiliations:** ^1^ Department of Cell Biology, Yale University School of Medicine, New Haven, CT, USA.; ^2^ Centre for Bioimaging Sciences, National University of Singapore, Singapore, Singapore.; ^3^ Department of Applied Physics, Yale University, New Haven, CT, USA.; ^4^ Department of Biomedical Engineering, Yale University, New Haven, CT, USA.

## Abstract

Self-organizing waves are observed in numerous biological systems and may encode spatial and temporal information for cellular organization in the absence of prepatterns. In mitotic mast cells, periodic cortical waves emerge before spindle assembly with wave periods that are proportional to cell size. Here, we investigate the mechanisms that govern cortical wave scaling and examine the consequence of wave perturbation on mitotic spindle size scaling. We find that the periods of mitotic waves are regulated by the turnover of phosphatidylinositol 3,4-bisphosphate [PI(3,4)P_2_] on the plasma membrane, which depends on inositol polyphosphate-4-phosphatase type II (INPP4B). Genetic depletion of INPP4B increases cortical wave period and spindle length. Intriguingly, we observed mitotic wave periods that tunes continuously during mitosis, indicating the existence of a fast, posttranslational regulatory mechanism for wave scaling. We further find that the regulation of mitotic waves on the plasma membrane is controlled by the sequestering of INPP4B and PI(3,4)P_2_ upon mitotic Golgi fragmentation. On the basis of these findings, we propose a cell size–sensing mechanism in which cortical waves act like sonar waves, adjusting their timing and propagation based on the shuttling of signaling proteins between the cell cortex and intracellular organelles. This rapid communication scheme allows the cell to adjust spindle scaling dynamically, ensuring accurate cell division.

## Teaser

Waves syncing the social network of mitosis.

## INTRODUCTION

Traveling waves influence diverse cellular processes, such as cell motility and polarity (*1*–*5*), cell cycle (*6*–*9*), and cell division (*10*–*12*). The reaction-diffusion model proposed by Turing (*13*) can explain a wide variety of complex patterns as generated by two interacting diffusible substances (activator and inhibitor) that have different lifetimes and diffusion rates. These mechanisms allow the system to self-organize and generate spatial and temporal patterns autonomously without pre-existing templates, or prepatterns.

In developmental biology, many reaction-diffusion systems display size-scaling capabilities (*14*–*22*). In its simplest form, the wavelength generated by interacting diffusible substances is primarily determined by their reaction rates and the square root of their diffusion constant, insensitive to system size (*23*). However, rate constants in biological systems are given by enzyme activity, and these, in turn, can be influenced by other substances such as activators and inhibitors, which may render rate constants size-dependent (*24*–*27*). For instance, the restriction on the quantity of locally produced activators, through the introduction of a saturation term, can lead to size regulation (*28*, *29*). In biology, this saturation term is sometimes referred to as a limited pool. Experimentally, relatively little is known about how traveling waves generated by subcellular reaction-diffusion systems may display size-scaling properties (*25*).

In our recent work, we discovered that active Cdc42 forms traveling waves on the surface of mast cells (rat basophilic leukemia, RBL-2H3 cells) (*30*). Formin-binding protein 17 (FBP17), an effector for Cdc42 that binds only to its active form, is an important regulator of these waves. FBP17 is an F-BAR domain containing protein that binds to negatively charged lipids (*31*, *32*), particularly phosphatidylinositol 4,5-bisphosphate and phosphatidylinositol (3,4,5)-trisphosphate (PI(3,4,5)P_3_) that are localized on the inner leaflet of the plasma membrane (*33*). In interphase, active Cdc42 and FBP17 waves are involved in membrane curvature propagation (*34*, *35*), endocytosis (*36*, *37*), and actin dynamics (*38*, *39*). In mitosis, active Cdc42 organizes into concentric target wave patterns. Target waves of Cdc42 oscillate with a period that scales with cell size: Larger cells exhibit longer oscillation periods (*12*). Inhibition of Cdc42 waves in metaphase reduces cell adhesion and prevents cells from entering anaphase, likely indicating that proper scaling of mitotic waves to the cell’s size is required for adhesion-dependent mitosis (*12*). However, precisely how Cdc42 waves display cell size–dependent periods and the function of mitotic Cdc42 waves are not known.

Cell size is an important regulatory factor during mitosis (*40*–*42*). During prometaphase and metaphase, a network of microtubule-interacting proteins forms a spindle within the cell to ensure accurate segregation of chromosomes into two daughter cells. The spindle assembly and positioning in relation to the chromosomes are important for proper attachment and alignment of chromosomes at the onset of anaphase. Proper control of the metaphase spindle size is crucial for the successful segregation of chromosomes during cell division (*43*, *44*). While it is known that the length of the metaphase spindle is proportional to cell size (about half of the cell size) in various cell types (*45*–*51*), the mechanisms that control this scaling relationship are not well understood. It is important to distinguish between control of spindle size and scaling of spindle size with cell dimensions. Perturbations of structural proteins that regulate microtubule polymerization, depolymerization, and sliding can influence spindle size (*44*, *52*–*54*). Such perturbations do not necessarily explain how spindle dimensions increase in proportion to cell size physiologically. The mechanisms that couple spindle length to cell size in the presence of an intact spindle assembly machinery remain mysterious (*55*–*57*).

In this work, we tested how mitotic Cdc42 waves displayed cell size–dependent properties and whether the scaling of mitotic Cdc42 waves can affect the assembly of the mitotic apparatus. We find that cortical wave dynamics can be dynamically tuned through phosphoinositide signaling, more specifically phosphatidylinositol 3,4-bisphosphate [PI(3,4)P_2_] turnover on the plasma membrane. Perturbing cortical wave dynamics by depleting inositol polyphosphate-4-phosphatase type II (INPP4B) also alters spindle length. In addition to plasma membrane, active Cdc42, PI(3,4)P_2_ and INPP4B are localized intracellularly on Golgi-derived membrane, and mitotic waves on the plasma membrane are acutely sensitive to mitotic Golgi fragmentation. Collectively, our results indicate that mitotic Cdc42 waves respond to dynamic communication between plasma membrane and intracellular membrane, mediated by signaling proteins that shuttle between them. We propose that the size-dependent mitotic wave period originates from a finite total pool of plasma membrane and intracellular membrane, resulting in a competition between Golgi-derived vesicles and the cell surface that links spindle length to cell size.

## RESULTS

### Mitotic waves are regulated by Cdk1 but do not depend on it for their size-scaling properties

**Fig. 1.**
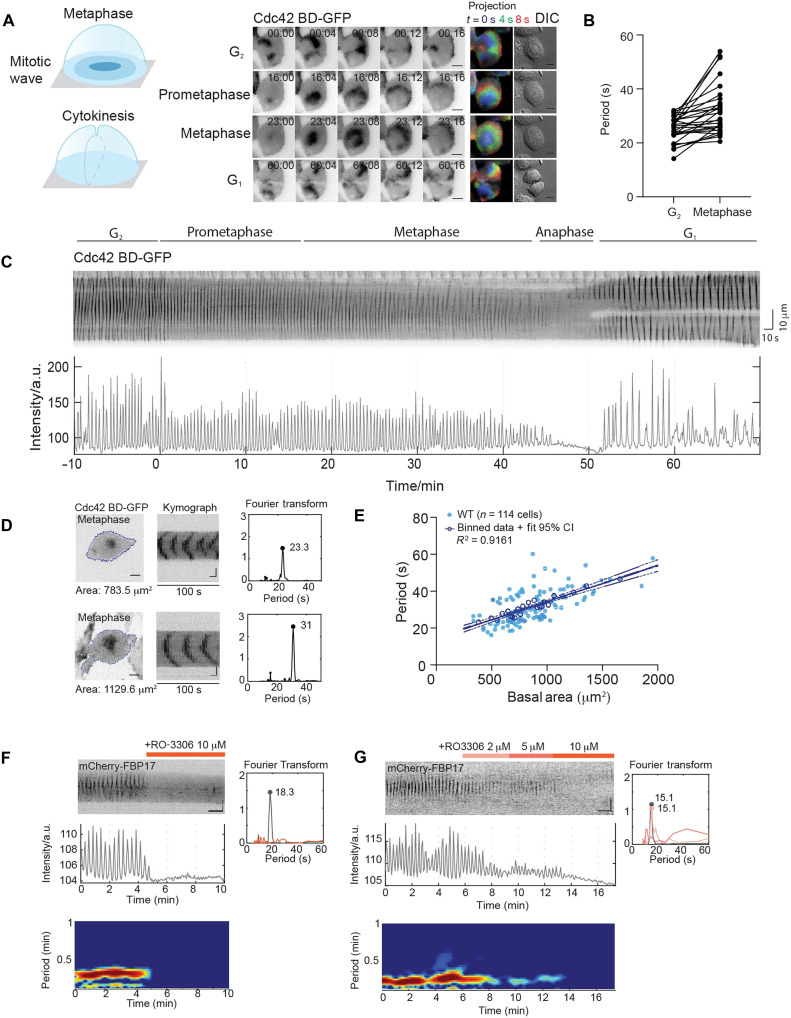
Scaling of mitotic wave period to cell size is not determined by Cdk1 mitotic kinase activity. (**A**) Schematic of mitotic wave in adhesion-dependent mitosis (left); representative TIRF and differential interference contrast (DIC) images of RBL-2H3 mast cells expressing Cdc42 BD-GFP (active Cdc42 biosensor) during different stages of mitosis (right). Pseudocolored time projection images for each phase was generated to illustrate the difference between traveling wave (G2 and G1) and target waves (prometaphase and metaphase). (**B**) Pairwise quantification of wave periods of the same cell at G2 and metaphase (*n* = 28 cells). (**C**) Kymograph (top) and intensity plots (bottom) of cell in (A). (**D**) Representative TIRF images, corresponding kymographs and oscillation period analysis of mitotic waves (via Fourier transformation of intensity profile) in cells with different sizes expressing Cdc42 BD-GFP. Blue dashed lines indicate basal area measurements. (**E**) Scatterplot of mitotic wave periods against mitotic cell size (*n* = 114 mitotic cells). Filled circles indicate individual single-cell data points, and open circles indicate binned data (later referred to as wave period scaling plot). Solid line shows simple linear regression fitting of data. The dashed lines show 95% confidence interval (CI). *R*^2^, coefficient of determination. (**F** and **G**) Kymograph, intensity plot, wavelet analysis (left), and Fourier transformation (right) before (gray line) and after (colored) of cells expressing mCherry-FBP17 treated with single (F) and multiple doses (G) of Cdk-1 inhibitor RO-3306. Time in (B), minutes:seconds. Scale bar, 10 μm. For kymographs, horizontal scale bar represents 10 s; vertical scale bar represents 10 μm.

Mitotic cells undergo extensive membrane and cytoskeletal remodeling, resulting in observable cell shape changes, generally rounding up during mitosis and flattening as they exit mitosis. Mitotic RBL cells become rounder than during interphase but remain largely attached to the substrate, allowing visualization of mitotic cortical dynamics using total internal reflection fluorescence (TIRF) microscopy. With a fluorescent biosensor that translocates to the plasma membrane upon binding active Cdc42 [active Cdc42 binding domain, Cdc42 binding domain (BD)–green fluorescent protein (GFP)], we observed that active Cdc42 forms traveling waves on the cell surface that switch depending on the mitotic stages. During metaphase, there is a transition from traveling waves to target waves. Target waves stop in anaphase and then transition back to traveling waves upon mitotic exit (Fig. 1A and movie S1). Both traveling waves and target waves display regular oscillations. We also observed an increase in oscillation period from interphase cells (G2 phase) to mitosis before nuclear envelope breakdown (*n* = 28 cells) (Fig. 1B), illustrated in kymographs and intensity plots (Fig. 1C and fig. S1A).

Using cell adhesion area as a measure for cell size, we found a positive correlation between mitotic wave period and cell size (*n* = 114 cells, 25 experiments). This is quantitatively illustrated in the representative kymographs of a small and big cell with different wave periods (Fig. 1D and movie S2). Fourier transform analysis of Cdc42 mitotic waves showed that periods are shorter in smaller cells and longer in larger cells (Fig. 1D). The scaling relationship is illustrated by a scatterplot of mitotic wave period versus mitotic basal area, with a statistically significant positive correlation between these two variables (Fig. 1E). In contrast, cells in G_2_ phase did not display such a scaling relationship (fig. S1B).

The scaling of Cdc42 waves with cell size during mitosis, therefore, suggests that, while the formation of cortical waves is not cell cycle dependent, their oscillation period is subject to mitosis-specific regulation. To determine whether these mitotic wave periods are regulated by mitotic kinases, we perturbed mitotic wave with an acute treatment of a Cdk1 inhibitor, RO-3306. We found that 10 μM RO-3306 acutely abolished cortical waves (Fig. 1F). A lower dosage of RO-3306 (2 to 5 μM) reduced the amplitude of mitotic waves, but not their periods (Fig. 1G). This suggests that, while mitotic wave amplitudes are regulated by Cdk1, the changes in wave periodicity that we observed during mitosis and their scaling with cell size are not regulated by phosphorylation of wave proteins by Cdk1.

### Mitotic wave period is regulated by the degradation rate of PI(3,4)P_2_

**Fig. 2.**
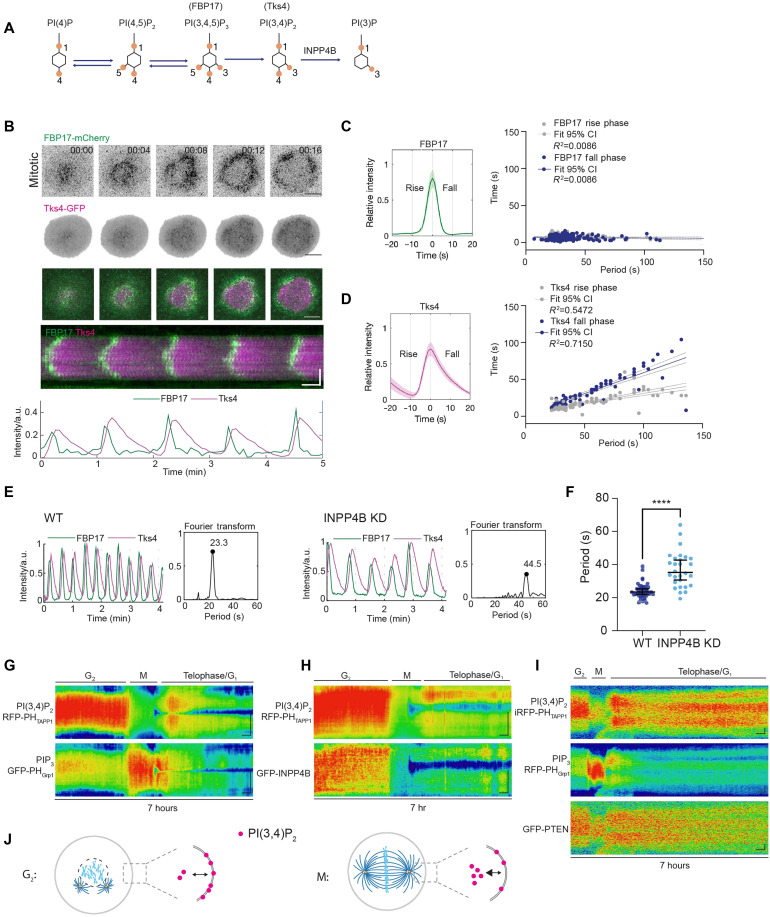
Mitotic wave period is controlled by INPP4B-dependent regulation of PI(3,4)P_2_ turnover. (**A**) Schematic illustrating phosphoinositide metabolism pathway, with FBP17 and Tks4 as effector for PI(3,4,5)P_3_ and PI(3,4)P_2_, respectively. (**B**) Representative montage, kymographs, and intensity plots of a mitotic cell coexpressing FBP17-mCherry and Tks4-GFP. (**C** and **D**) Average profiles (left) and scatterplot (right) of the rise and fall phases of FBP17-mCherry (C) and Tks4-GFP (D). Solid lines in average profiles represent the mean intensities of the profile, and the shaded region represents the SDs. Dark blue circles in scatterplots indicate data from fall phase, and gray circles indicate data from rise phase. *R*^2^, coefficient of determination. (**E**) Representative intensity plots and period analysis of WT and INPP4B KD cells. a.u., arbitrary units. (**F**) Distribution of wave periods in WT and INPP4B KD cells (*N* = 6, *n* = 39 WT cells, *n* = 30 INPP4B KD cells; error bar, SD; ****P < 0.001, *t* test). (**G** to **I**) Mitotic-specific changes in lipids and metabolic enzymes at the plasma membrane. Cells were imaged by TIRF microscopy every 2 min for 7 hours, capturing dynamics before and after mitotic entry. Representative kymographs are shown. (G) A cell expressing RFP-PH_TAPP1_, sensor for PI(3,4)P_2_ and GFP-PH_Grp1_, sensor of PI(3,4,5)P_3_ (*N* = 8, *n* = 25 of 26 mitotic cells); (H) a cell expressing RFP-PH_TAPP1_, sensor for PI(3,4)P_2_ and GFP-INPP4B (*N* = 9, *n* = 27 of 30 mitotic cells); (I) a cell expressing RFP-PH_TAPP1_, sensor for PI(3,4)P_2_, and iRFP-PH_Grp_, sensor of PI(3,4,5)P_3_ and GFP-PTEN (*N* = 2, *n* = 2 of 2 cells). (**J**) Schematic illustrating reduction of PI(3,4)P_2_ and INPP4B localization at the plasma membrane as cells proceed from G_2_ to mitosis. For images, scale bar, 10 μm. For kymographs, horizontal scale bar represents 10 s; vertical scale bar represents 10 μm.

Traveling waves arise from spatially coupled positive and negative regulators. In particular, oscillations at each location occur when the inhibitor has a longer time constant than the activator; under this condition, the inhibitor lifetime sets the oscillation timescale. Our previous work showed that interphase cortical waves mediated by active Cdc42 and FBP17 are coupled to cycles of phosphoinositide turnover on the plasma membrane (*58*). Specifically, PI(4,5)P_2_ and PI(3,4,5)P_3_ recruit active Cdc42 and FBP17 (Fig. 2A), and their oscillations are mediated by the sequential action of phosphoinositide 3-kinase (PI3K) and phosphoinositide 5-phosphatase (*58*). Overexpression of PI3K or its inhibition (using wortmannin or PI3Kδ specific inhibitor Cal-101) increased or decreased the oscillation period, respectively (*58*). However, both PI(4,5)P_2_ and PI(3,4,5)P_3_ are short-lived relative to the period of cortical oscillations and show little variabilities when the oscillation period changes, suggesting that their lifetimes do not directly determine the tuning of cortical oscillation period.

We therefore examined the lifetime and turnover of their downstream product, PI(3,4)P_2_, to identify the rate limiting step controlling wave periodicity. To monitor PI(3,4)P_2_ dynamics, we coexpressed Tks4, a PI(3,4)P_2_-binding protein, with FBP17 in the same cell. FBP17 waves preceded Tks4 waves in both interphase (fig. S2, A and B, and movie S3) and mitotic cells (Fig. 2B and movie S4) (*n* = 51 interphase cells, 16 mitotic cells). Intensity profiles revealed that the lifetime of Tks4 consistently correlated with the interspike interval of FBP17 oscillations. Although both proteins exhibited a sharp rise, Tks4 declined more gradually than FBP17 and persisted until the onset of the next FBP17 cycle exhibiting an activator-inhibitor relationship.

When cells with varying oscillation periods were compared, the slope of the Tks4 rise phase was similar across cells, whereas the slope of the Tks4 decay phase varied (fig. S2, C and D). Neither the rise nor the decay phase of FBP17 correlated with oscillation period (Fig. 2C and fig. S2E). In contrast, the rise phase of Tks4 showed a weak correlation with oscillation period, while the decay phase of Tks4 significantly correlated with oscillation period (Fig. 2D). These results indicate that variability in mitotic wave period is primarily determined by the dissociation of Tks4 from the plasma membrane, consistent with PI(3,4)P_2_ degradation being the rate-limiting step.

### INPP4B-dependent PI(3,4)P_2_ degradation mediates cortical wave period tuning

Because Tks4 is a PI(3,4)P_2_ effector protein, we next examined the role of INPP4B, a lipid phosphatase that degrades PI(3,4)P_2_ by converting PI(3,4)P_2_ to PI(3)P, in regulating mitotic waves. We generated INPP4B knockdown (KD) cells using short hairpin RNA (shRNA) and monitored their wave properties. Notably, INPP4B KD increased the cortical wave period from 24.2 ± 4.7 s (*n* = 39 cells) to 40.7 ± 17.7 s (*n* = 30 cells) (Fig. 2, E and F). The prolonged wave periods in KD cells were associated with a slower PI(3,4)P_2_ decay phases (Fig. 2E). These results suggest that INPP4B modulates the turnover rate of PI(3,4)P_2_ in mitosis and thereby controls cortical wave periodicity.

Although genetic depletion of INPP4B was sufficient to change mitotic wave period, this does not necessarily imply that size-dependent control occurs at the level of gene expression. If size-dependent scaling of mitotic waves was driven by intrinsic variation in INPP4B expression, a similar scaling relationship should be observed in interphase cells, which we did not find (fig. S1B). We therefore decided to investigate whether the level of PI(3,4)P_2_ and INPP4B was regulated posttranslationally, such that their recruitment to the plasma membrane changed during mitosis.

To quantitatively assess PI(3,4)P_2_ levels in single cells, we used TIRF imaging at reduced acquisition interval (every 2 min), enabling continuous imaging of individual cells for up to 7 hours. We monitored plasma membrane PI(3,4)P_2_ levels in wild-type (WT) cells throughout the cell cycle using red fluorescent protein (RFP)–PH_TAPP1_ as a sensor. We observed a global reduction of PI(3,4)P_2_ at mitotic onset (*n* = 27 of 30 mitotic cells, nine experiments) (Fig. 2G and fig. S3A). PI(3,4)P_2_ levels recovered upon mitotic exit, indicating that the reduction was unlikely due to photobleaching. To determine whether the decrease in PI(3,4)P_2_ levels during mitosis resulted from availability of its upstream source PI(3,4,5)P_3_, we imaged GFP-PH_Grp1_ [PI(3,4,5)P_3_ sensor]. Unexpectedly, PI(3,4,5)P_3_ levels increased at mitotic onset when PI(3,4)P_2_ decreased (*n* = 25 of 26 mitotic cells, eight experiments) (Fig. 2G), indicating that the reduction in PI(3,4)P_2_ was not due to limited PI(3,4,5)P_3_ supply. To exclude the possibility that these changes reflected fluctuations of membrane within the TIRF field, we cotransfected an iRFP cytosolic control with RFP-PH_TAPP1_ and GFP-PH_Grp1_. While PI(3,4)P_2_ decreased and PI(3,4,5)P_3_ increased at mitotic onset, iRFP control remained unchanged throughout the cell cycle (*n* = 3 of 3 cells, two experiments) (fig. S3B).

To examine INPP4B dynamics, we cotransfected RFP-PH_TAPP1_ and GFP-INPP4B in the same cells. Both PI(3,4)P_2_ and INPP4B levels decreased simultaneously at mitosis entry (*n* = 27 of 30 cells, nine experiments) (Fig. 2H). This reduction was specific to INPP4B: Neither PI(3,4,5)P_3_ nor phosphatase and tensin homolog (PTEN), a 3-phosphatase that metabolizes 3′ phosphate of PI(3,4,5)P_3_ and PI(3,4)P_2_, showed similar changes in the same cell (*n* = 2 of 2 cells, two experiments) (Fig. 2I). These findings support a model in which reduced recruitment of INPP4B to the plasma membrane during mitosis slows PI(3,4)P_2_ turnover and prolongs the period of mitotic waves (Fig. 2J).

Notably, the effect of INPP4B on PI(3,4)P_2_ levels is counterintuitive. As a phosphatase that degrades PI(3,4)P_2_, reduced INPP4B would be expected to increase PI(3,4)P_2_ levels. However, because lipid synthesis and degradation are tightly coupled within an oscillatory cycle, slowing PI(3,4)P_2_ turnover lengthens the oscillation period and reduces the number of synthesis cycles as well, ultimately decreasing the average PI(3,4)P_2_ level.

### Scaling of mitotic waves is coupled to mitotic spindle scaling

**Fig. 3.**
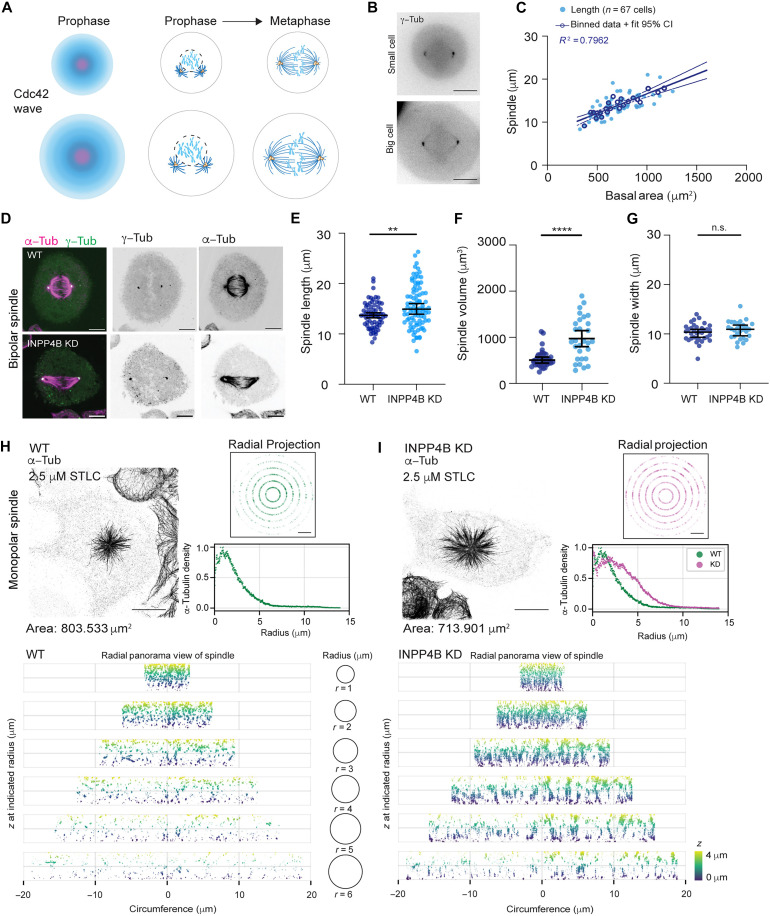
INPP4B KD increases mitotic spindle sizes by increasing microtubule density. (**A**) Schematics illustrating mitotic wave and scaling of spindle size in metaphase. (**B**) Representative immunofluorescence images of spindle pole in mitotic cells stained with γ-tubulin. (**C**) Scatterplot of spindle size (γ-tubulin separation) versus basal area of mitotic cells based on reconstruction of 3D spinning disk confocal images (*N* = 11, *n* = 67 cells). Filled circles indicate individual data points, and open circles indicate binned data points. Solid lines show simple linear regression fitting of data. The dashed lines show 95% confidence interval (CI). (**D**) Representative immunofluorescence images of mitotic spindles in WT (top) and INPP4B KD (bottom) cell stained with α-tubulin and γ-tubulin antibody. (**E** to **G**) Distribution of spindle length (E), spindle width (F), and spindle volume in WT and INPP4B KD cells (E: *N* = 11, *n* = 67 WT cells; *N* = 4, *n* = 89 INPP4B KD cells; error bar, SD; **P < 0.01, *t* test; F: *N* = 4, *n* = 35 WT cells; *N* = 4, *n* = 29 INPP4B KD cells; error bar, SD; ****P <0.0001, *t* test; G: *N* = 4, *n* = 35 WT cells; *N* = 4, *n* = 29 INPP4B KD cells; error bar, SD; *P* = 0.2194, *t* test; n.s., not significant). (**H** and **I**) Quantification of microtubule density by super-resolution imaging of monopolar spindles in WT (H) and INPP4B KD (I) cells. Top left: Representative image from single-molecule DNA-PAINT imaging of monopolar spindles. Top right: Radial projection of microtubule density at six different radius (1 to 6 μm) and intensity profile of α-tubulin density as a function of radius. Bottom: Radial panorama view of DNA-PAINT image at the indicated radial distance (*N* = 4, *n* = 17 WT cells, *n* = 15 KD cells). WT (H) and KD (I) are presented as size-matched pairs to enable direct comparison of radial density decay profiles. For images, scale bar represents 10 μm. For radial projections in (H) and (I), scale bar represents 1 μm.

Because mitotic waves are observed before spindle assembly and microtubules self-organize into a bipolar spindle of appropriate sizes during metaphase, we hypothesized that mitotic waves initiate in early mitosis to facilitate proper spindle assembly (Fig. 3A). We first validated the relationship between spindle size and cell size in RBL cells by quantifying the scaling of spindles size relative to cell size. We used mitotic cell adhesion area as a proxy for cell size and the centrosome marker γ-tubulin to measure spindle length (Fig. 3B). A scatterplot of spindle size versus mitotic basal area revealed a positive scaling correlation between these two parameters (Fig. 3C).

Given that both mitotic wave periodicity and spindle size scale with mitotic cell size, we next tested whether altering wave periods affect spindle size. We compared spindle sizes between WT and INPP4B KD cells. Three-dimensional (3D) confocal immunofluorescence imaging of α-tubulin, centrosome markers (γ-tubulin and pericentrin), and 4′,6-diamidino-2-phenylindole (DAPI) staining was used to measure spindle length, width, and volume (Fig. 3D). INPP4B KD resulted in a notable increase in mitotic spindle size compared with WT cells. Specifically, spindle length increased from 13.3 ± 2.4 μm in WT cells (*n* = 67 cells) to 15.5 ± 4.2 μm in KD cells (*n* = 89 cells, *P* < 0.01) (Fig. 3E). Consistent with this increase in length, spindle volume was also significantly elevated in KD cells (WT volume of 533.3 ± 194.8 μm^3^, *n* = 35 cells; INPP4B KD volume of 973.1 ± 428.2 μm^3^, *n* = 29 cells; *P* < 0.0001) (Fig. 3F). In contrast, spindle width did not change significantly (WT width of 10.3 ± 1.8 μm, *n* = 35 cells; INPP4B KD width of 10.8 ± 1.8 μm, *n* = 29 cells; *P* = 0.2194) (Fig. 3G), suggesting that cortical wave perturbation preferentially affects longitudinal spindle scaling while preserving radial spindle organization.

To further dissect the mechanism underlying spindle elongation, we examined whether INPP4B KD affects the size of monopolar spindles induced by the Eg5 inhibitor *S*-trityl-l-cysteine (STLC), which blocks motor-driven sliding of antiparallel microtubules. Consistent with our findings in bipolar spindles, monopolar spindles were longer in INPP4B KD cells compared with WT cells (Fig. 3, H and I, and fig. S4). We used DNA-PAINT–based single-molecule-localization microscopy imaging to qualitatively assess microtubule organization. Cross-sectional panoramic views were generated by measuring α-tubulin single-molecule localization count along circumferential arcs at increasing radial distances from the monopolar spindle pole. In both WT and KD cells, monopolar spindle exhibited a decrease in α-tubulin density with increasing distance from the pole (Fig. 3, H and I). Density was quantified by measuring average localization counts as a function of radial distance from the monopolar spindle center. However, this radial decay was substantially attenuated in INPP4B KD cells of comparable size (*n* = 17 cells for WT and *n* = 15 cells for KD, four experiments) (Fig. 3I and fig. S4). As a result, microtubules remained denser at distal regions in KD cells.

Together, these findings indicate that perturbation of wave dynamics via INPP4B depletion increases spindle size, supporting a functional coupling between mitotic cortical wave dynamics and spindle size control. Furthermore, they suggest that the increase in spindle size arises primarily from a slowing of the radial decay of microtubule density, thereby promoting spindle elongation.

### Mitotic tuning of cortical wave periodicity continues into metaphase

**Fig. 4.**
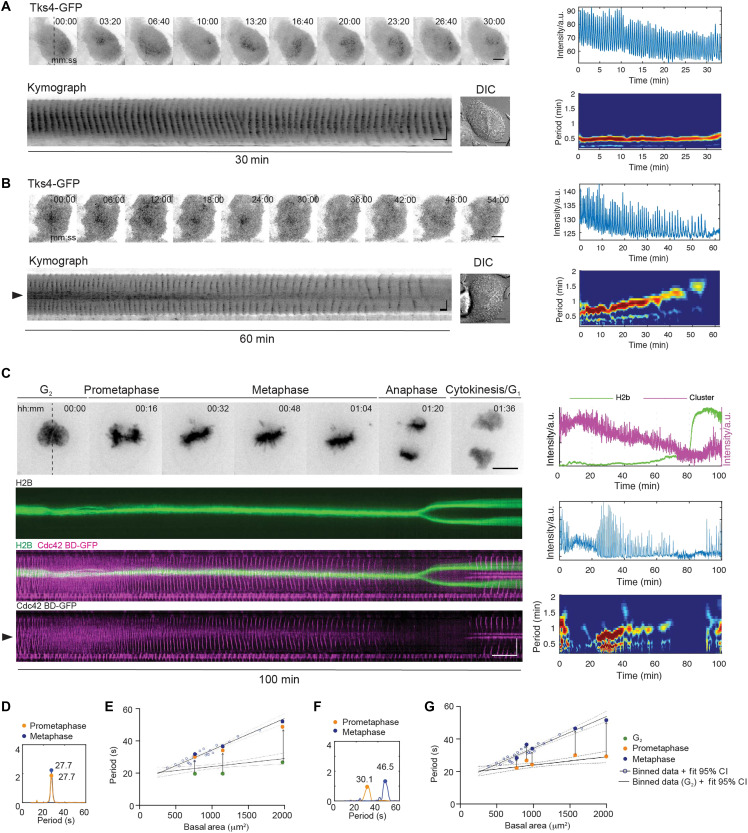
Dynamic tuning of mitotic wave period could occur during metaphase and is associated with the disappearance of an intracellular cluster that sequesters wave markers. (**A** and **B**) Representative TIRF montages, kymograph, and DIC image (left), corresponding intensity profile with wavelet analysis (right) of mitotic cell expressing Tks4-GFP with constant wave period during metaphase (A) or dynamic wave period (B). Dashed lines indicate regions used to generate kymographs. Clusters (indicated with arrowhead) are visible only in cells with dynamic wave periods and disappear with time (B). (**C**) Quantification of mitotic wave in a cell expressing H2B-RFP and Cdc42BD-GFP. Left: Montage and kymograph. Right: Intensity profile of cluster (ROI at the center of the cell, indicated with arrowhead), wave (ROI off the center), and H2B (ROI centered at daughter nucleus) and wavelet analysis of the Cdc42 wave. Dashed line indicates region used to generate kymographs. Clusters disappear completely at anaphase onset. a.u., arbitrary units. (**D**) Period analysis of a representative cell expressing Tks4-GFP, showing identical wave periods in prometaphase and metaphase. (**E**) Cortical wave periods for three cells tracked from interphase/G_2_ (green dot) to prometaphase (orange dot) to metaphase (blue dot) overlaid on the scaling plot in Fig. 1E. These cells reach oscillation period predicted by cell sizes early in prometaphase. (**F**) Period analysis of a representative cell expressing Tks4-GFP, showing that its wave period in metaphase is slower compared to that in prometaphase. (**G**) Cortical wave periods for five cells tracked from prometaphase (orange dot) to metaphase (blue dot) overlaid onto scaling plot in Fig. 1E. These cells dynamically tune their period until they reach the scaling property. For images, scale bar represents 10 μm. For kymographs, horizontal scale bar represents 1 min, except (C) where horizontal scale bar represents 5 min; all vertical scale bars represent 10 μm.

To identify mitotic mechanisms that may sequester regulators of cortical waves, such as PI(3,4)P_2_ and INPP4B, we examined the G_2_-M transition and the establishment of wave scaling. Two distinct behaviors emerged. In the majority of cells (86%, 24 of 28 cells), cortical waves displayed a mitotic period that remained constant throughout mitosis, as confirmed by intensity plots and wavelet analysis (Fig. 4A, fig. S5A, and movie S5). In contrast, a minor population of cells (14%, 4 of 28 cells) exhibited active tuning of wave periodicity during mitosis, with period progressively increasing over time (Fig. 4B and fig. S5B). In these cells, kymograph of Tks4 revealed a nonoscillatory cluster of Tks4 that was present in the early mitosis but gradually dissipated as wave periods increased (Fig. 4B and fig. S4B). Notably, the presence of this nonoscillatory Tks4 cluster strongly correlated with active period tuning. Among 16 metaphase cells exhibiting the nonoscillatory pool, 12 showed dynamic tuning. Conversely, in cells lacking this pool, wave periods remained constant during mitosis (80%, 8 of 10 cells).

To further examine the temporal relationship between this nonoscillatory pool and mitotic progression, we imaged cortical waves in synchronized cells coexpressing chromosome marker RFP-H2B. Upon mitotic entry, a cluster of active Cdc42 appeared in the center of the cell (Fig. 4C). This cluster was distinct from cortical waves on the kymograph, persisted through metaphase and disappeared at the onset of anaphase, coincident with chromosome segregation (Fig. 4C). The reduction of Cdc42 cluster also correlated with a gradual increase in wave period, as shown in the wavelet analysis (Fig. 4C). In contrast to waves showing constant wave period that scaled with cell size in prometaphase (Fig. 4, D and E), waves that dynamically tuned their wave periods converged onto the same scaling curve later in metaphase (Fig. 4, F and G).

### Wave proteins localize to a Golgi-associated intracellular pool

**Fig. 5.**
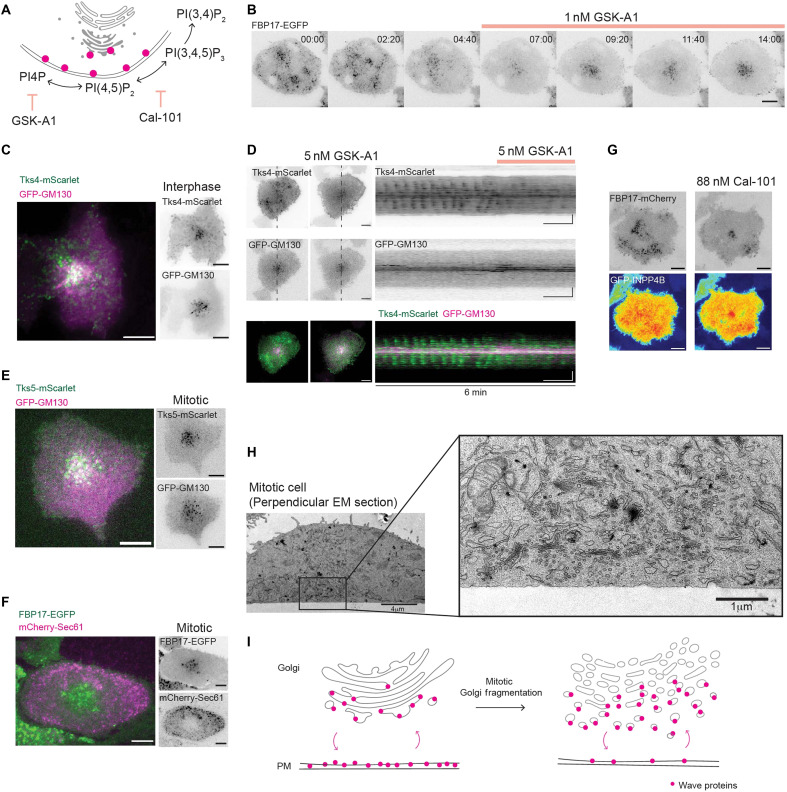
Intracellular cluster that sequesters wave regulators is related to Golgi. (**A**) Schematic illustrating the phosphoinositide metabolism pathway and the site of action for the indicated inhibitors. (**B**) Montages of cell expressing FBP17-EGFP before and after 1 nM GSK-A1 treatment. (**C**) Merged TIRF images of an interphase cell expressing Tks4-mScarlet and GFP-GM130. (**D**) TIRF images and kymograph of an interphase cell expressing Tks4-GFP and GFP-GM130(NLS-AA)-FM before and after treating with 5 nM GSK-A. Dashed lines indicate regions used to generate kymographs. (**E**) Merged TIRF images of a mitotic cell expressing Tks5-mScarlet and GFP-GM130. (**F**) Merged TIRF images of a mitotic cell expressing FBP17-EGFP and mCherry-Sec61. (**G**) TIRF images of an interphase cell expressing FBP17-mCherry and INPP4B-GFP before and after treating with 88 nM Cal-101. (**H**) Electron microscopy (EM) analysis of a mitotic cell (left) and the region close to the basal area (right). A population of vesicles is visible close to the adhering plasma membrane in close proximity to Golgi apparatus. (**I**) Graphical representation of the dynamic redistribution of wave proteins, including PI(3,4)P_2_ and INPP4B, between intracellular Golgi-derived membranes and the plasma membrane (PM). Upon Golgi fragmentation, the intracellular pool of wave proteins is dispersed and become below detection limit. For images, scale bar represents 10 μm. For kymographs, horizontal scale bar represents 1 min; all vertical scale bars represent 10 μm.

The persistence of an intracellular nonoscillatory pool in cells that continued to tune their wave periods in mitosis suggested the existence of a slow sequestration mechanism that affects wave regulators beyond mitotic entry. We first asked whether this pool resided at the plasma membrane or intracellularly. Acute inhibition of plasma membrane phosphoinositide dynamics using the PI4KIIIα inhibitor GSK-A1 (*59*) or the PI3Kδ inhibitor Idelalisib (Cal-101) (*58*) abolished cortical waves but revealed a persistent central cluster of wave proteins (Fig. 5, A and B, and movie S6). This cluster was detected with FBP17 and Tks4 (fig. S6, A to C). Tks5, which shares similar domain structures and functions with Tks4 but with a PX domain that binds to PI(3)P and less strongly to PI(3,4)P_2_, colocalizes with Tks4 and was enriched in the central pool (fig. S6D).

Resistance to plasma membrane lipid inhibition indicated that the nonoscillatory pool is unlikely on the plasma membrane, although it remained within the TIRF field. Colocalization analysis revealed proximity to Golgi marker GM130 (Fig. 5, C to E), mannosidase II, and TNG46 (fig. S6, F and G), but exclusion from the ER marker Sec 61 (Fig. 5F). We then investigated INPP4B distribution and dynamics upon wave inhibition with acute Cal-101 treatment and found its enrichment to the FBP17 colocalizing cluster (Fig. 5G and fig. S6E).

Because mammalian Golgi membranes undergo fragmentation during mitosis, generating dispersed vesicular intermediates (*60*–*63*), we speculate that the intracellular nonoscillatory pool corresponds to incompletely fragmented Golgi membranes. Detecting Golgi fragmentation by fluorescence is challenging because Golgi markers appear diffusive after vesiculation. Therefore, we conducted electron microscopy analysis of mitotic cells and observed a population of vesicles residing within 100 nm of the adhering plasma membrane, adjacent to partially vesiculated Golgi mini stacks (Fig. 5H).

On the basis of the presence of incompletely fragmented Golgi mini stacks in early mitosis that are in close proximity to the plasma membrane and their shared affinity for wave proteins, we propose that mitotic Golgi fragmentation increases the surface-to-volume ratio of intracellular membrane, generating a membrane sink for PI(3,4)P_2_. As Golgi stacks disassemble into clustered remnants (cluster signal in TIRF) and smaller vesicles (undetectable by TIRF), wave-associated proteins dynamically partition between the plasma membrane and Golgi-derived membranes. Expansion of vesiculated Golgi membrane reservoir increases sequestration of PI(3,4)P_2_ and INPP4B, consequently reducing their cortical abundance and tuning wave periodicity (Fig. 5I).

### Perturbation of Golgi fragmentation controls mitotic wave scaling

**Fig. 6.**
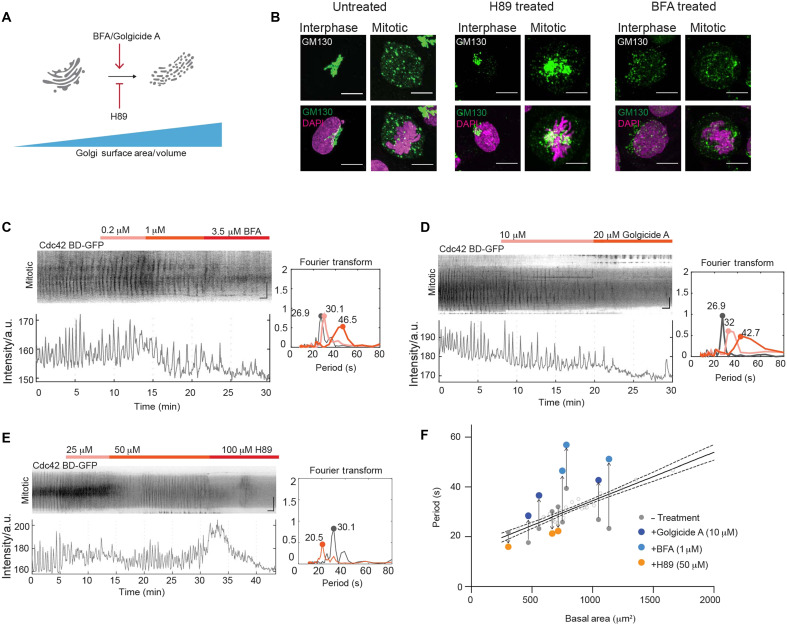
Perturbation of mitotic Golgi fragmentation impact wave scaling. (**A**) Schematic depicting the action of inhibitors on Golgi morphological state. (**B**) Immunofluorescence analysis of Golgi marker GM130 (green) and DNA (magenta) in the indicated cell cycle stage in untreated condition, after 100 mM H89 treatment or after 3.5 μM BFA treatment. (**C** to **E**) Representative kymograph (top), intensity profile (bottom), and quantification of wave periods from representative mitotic cells treated with the indicated concentration of BFA (C), Golgicide A (D), and H89 (E). Color line on the Fourier transformation plots corresponds to the indicated concentration of drugs (H89: *N* = 3, *n* = 3 mitotic cells; BFA: *N* = 3, *n* = 3 mitotic cells; Golgicide A: *N* = 2, *n* = 3 mitotic cells). a.u., arbitrary units. (**F**) Overlay of wave periods before and after indicated Golgi state perturbation on wave period scaling plot from Fig. 1E.

To test whether sequestration of wave-tuning proteins by the intracellular pool of Golgi-derived vesicles could impact wave scaling, we used three pharmacological approaches to perturb Golgi remodeling during mitosis (Fig. 6A). To inhibit Golgi fragmentation and reduce mitotic Golgi surface-to-volume ratio, we used H89 treatment. During mitosis, H89 inhibits Arf1 guanosine triphosphatase (GTPase) inactivation, blocking the disassembly of Golgi, resulting in inhibition of Golgi vesiculation (*64*–*66*). In contrast, brefeldin A (BFA) treatment causes persistent inactivation of Arf1 GTPase and induces Golgi disassembly, resulting in vesiculation (*67*–*70*). We also treated cells with Golgicide A, which more specifically cause Golgi fragmentation (*71*). GM130 staining confirmed modulation of Golgi architecture under each condition (Fig. 6B).

Acute BFA (Fig. 6C and fig. S6A) or Golgicide A (Fig. 6D and fig. S6B) treatment of mitotic cells resulted in longer mitotic wave periods in a dose-dependent manner. In contrast, inhibition of Golgi fragmentation by H89 resulted in shorter wave periods (Fig. 6E and fig. S6C). Relative to untreated scaling relationships, H89 treatment induced underscaling (three of the three cells, three experiments), whereas BFA (three of the three cells, three experiments) and Golgicide A treatment (three of the three cells, two experiments) induced overscaling (Fig. 6F). These results support a model that cortical wave periods are acutely sensitive to the morphological state of Golgi membranes.

## DISCUSSION

**Fig. 7.**
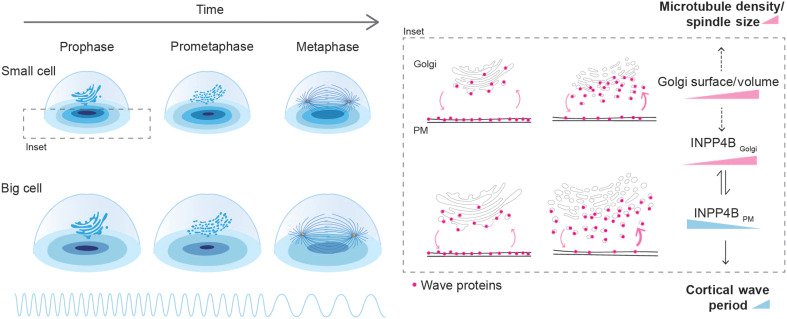
Schematic of the dynamic shuttling model for scaling of mitotic wave period and spindle size. Top row: Mitotic fragmentation increases the surface-to-volume ratio of the Golgi, enhancing sequestration of INPP4B. This reduces INPP4B at the plasma membrane (PM), slows PI(3,4)P_2_ turnover, and lengthens the mitotic wave period relative to interphase. Bottom row: The same process in a larger cell. Assuming a larger Golgi volume, fragmentation sequesters proportionally more wave components, leading to a longer wave period compared to a smaller cell. In this model, wave period serves as a readout of Golgi morphological state. Mitotic Golgi fragmentation, in turn, modulates microtubule nucleation and density away from the centrosome, thereby controlling spindle length and its scaling with cell adhesion area.

Through quantitative analysis of emergent cortical Cdc42 wave dynamics, we identify the mechanisms that tune wave periodicity and demonstrate how this tuning impacts mitotic machinery. We demonstrate that cell-size scaling of cortical wave periodicity is determined by INPP4B-dependent turnover of PI(3,4)P_2_ and that modulation of PI(3,4)P_2_ is functionally coupled to spindle size scaling. Perturbation of INPP4B alters spindle length through changes in microtubule density, thereby linking cortical wave dynamics with the regulation of spindle architecture. We further show that periodic traveling waves of Cdc42 during mitosis are regulated by membrane exchange between the plasma membrane and the Golgi through actively shuttling of phosphoinositide-binding signaling proteins. In this model, Golgi fragmentation at mitotic entry and throughout mitosis increases intracellular membrane surface area, redistributes wave components away from the plasma membrane, and tunes cortical wave dynamics (Fig. 7). In larger cells, assuming organelle volume scales with cell volume, fragmentation of a larger Golgi generates proportionally more membrane surface area, creating a larger membrane sink that sequesters wave components, including PI(3,4)P_2_ and INPP4B, from the plasma membrane, thereby achieving size-dependent tuning of mitotic wave period. This integrated system constitutes a rapid, nongenetic mechanism that senses organelle state in real time and modulates the size of the mitotic machinery (Fig. 7).

A key distinction of the size-scaling mechanism proposed here is its rapid timescale. Most current scaling models emphasize genetic regulation; for example, size-independent protein expression can yield concentrations inversely proportional to cell size (*72*–*74*). While our results do not exclude such regulation, the kinetics of mitotic wave tuning observed here are substantially faster than would be expected from transcriptional or translational control.

Dynamic partitioning of regulatory proteins between the plasma membrane and cytosol has previously been proposed to contribute to spindle scaling. Cell-free reconstitution studies have suggested that spindle size is sensitive to boundary conditions (*56*, *75*, *76*). In one study, importin-α, an inhibitor of spindle assembly, was shown to partition between the plasma membrane and cytosol, with small cells sequestering more importin on the plasma membrane, leading to a larger spindle size (*57*). This model predicts that larger cells have proportionally smaller spindles. For a plasma membrane surface-to-volume ratio model to explain spindle scaling, however, the plasma membrane must sequester a microtubule nucleation or polymerization activator (*49*, *56*). Another important assumption is that larger cells have a smaller surface-to-volume ratio compared with small cells. Recent work challenges this assumption, suggesting that small and large cells maintain similar surface-to-volume ratios because plasma membrane in large cells generated more membrane folds (*77*). Our model bypasses this assumption by proposing that the site of surface-to-volume ratio control is intracellular. Although little is known about surface-to-volume ratio for Golgi in small versus large cells, the increased sequestering capability conferred by mitotic Golgi fragmentation does not require any specific regulation beyond the average scaling relations. Instead, fragmentation intrinsically increases intracellular membrane surface area, providing a size-dependent membrane sink for wave components.

An oscillation-based model also circumvents limitations associated with the classic “limited-component” hypothesis, in which spindle size scaling is regulated by one or a few core components that limit spindle growth or net assembly (*78*). While this model explains certain scaling behaviors, it faces conceptual and practical challenges for size control of organelles with multiple copies (*79*). Limited-pool mechanisms often require accurate measurement of protein concentrations in low-abundance regimes, where stochastic fluctuations and measurement noise are substantial (*80*). A regulatory control mechanism through periodic dynamics can overcome this problem by improving the precision of concentration measurement through time averaging (*80*).

Although not direct tested here, Golgi-derived membrane could provide a mechanistic link between cortical dynamics and microtubule nucleation, thereby influencing spindle size. A recent study reported that spindle scaling is achieved primarily through changes in filament number rather than filament length (*56*). Mechanisms regulating nucleation of microtubules, especially from the membrane, are still poorly understood, but the Golgi has emerged as an important noncentrosomal microtubule-organizing center (*81*–*83*). Small GTPase Cdc42 has been shown to act not only at the plasma membrane but also at the Golgi (*84*). Cdc42-interacting protein 4, a paralog of FBP17, has been reported to interact with AKAP350, a microtubule nucleator, at the Golgi (*85*, *86*). In this model, mitotic waves can tip the balance between the plasma membrane and intracellular pool of microtubule nucleating proteins and influence microtubule nucleation at the minus end. Using super-resolution imaging, we show that the decay of microtubule density as a function of distance from the centrosome is greatly reduced in INPP4B KD cells, consistent with enhanced noncentrosomal microtubule nucleation or branching. Alternatively, wave proteins could regulate the stability of microtubule at the plus end. We recently showed that FHDC1, a microtubule and actin binding protein, colocalizes with microtubules and oscillates with FBP17 in the cortical region (*38*). Disruption of cortical waves inhibited FHDC1 and cortical microtubule dynamics. Thus, cortical waves could directly regulate the dynamics or stability as well as nucleation of microtubule, all of which could contribute to spindle assembly.

These findings expand cortical waves from a purely surface-restricted phenomenon to an integrated systems level readout that encodes the mitotic state of intracellular membrane. This raises the question of why sensing at the cell surface would be coupled to spindle size control. In mast cells, which remain adherent during mitosis, inhibition of Cdc42 waves induces cell rounding and prevents anaphase entry, suggesting that adhesion is required for their mitosis. In contrast to conventional mitosis, where cells round up, lose adhesion, and undergo symmetric contractility, adhesion-dependent mitosis is polarized and proceeds through unilateral furrowing. This mode of division plays important roles in tissue architecture and anchorage-dependent proliferation of stem cells (*87*, *88*), immune cells (*89*–*91*), and developing embryos (*92*, *93*). Classic experiments by Rappaport (*94*–*99*) demonstrated that eccentric displacement of the mitotic apparatus in sand dollar eggs converts symmetric furrowing into unilateral furrowing, indicating that, while not all cells normally divide in this manner, all cells have the capacity to respond to changes in the geometric relationship between the mitotic apparatus and the cortex. Adhesion-dependent mitosis requires a symmetry-breaking event before anaphase onset to orient cell division. In this context, Cdc42-dependent size sensing may function analogously to a polarity-establishing mechanism that couples cortical organization to spindle positioning. Propagating Cdc42 waves during mitosis may therefore represent an active mode of environmental sensing that integrates adhesion, geometry, and spindle dynamics.

More broadly, our work leverages dynamical patterns as a sensitive readout of the underlying molecular circuitry to dissect interactions among multiple subcellular machineries, which are otherwise difficult to resolve through genetic perturbations. It remains to be seen whether the wave tuning model that we propose here could be generalized in other systems for size sensing and scaling. Interphase cortical waves have been widely observed in many cellular systems, such as *Dictyostelium*, *Physarum polycephalum*, mast cells, neutrophils, T cells, B cells, macrophages, dendritic cells, fibroblasts, as well as in epithelial cells during tissue morphogenesis (*100*–*103*). A few examples of mitotic cortical waves have been reported, including Rho waves in *Xenopus* embryos and starfish oocytes (*10*, *11*) and actin waves in fibroblasts (*104*–*106*), but size-scaling properties of these patterns have not been tested in most contexts. In giant interphase cell of *Dictyostelium discoideum*, actin waves show size-independent wavelength (*107*). In developing *Xenopus* embryos, a trend in the decrease in the cortical wave periods and their variabilities could be observed as development progresses (*108*). In growing *Escherichia coli*, an oscillating wave of Min system does not have size-dependent oscillation period but displays steeper phase gradient instead (*109*). We are only beginning to uncover how the emergent properties of these mesoscale patterns are harnessed in physiological contexts. Nonetheless, such patterns offer a powerful conceptual and quantitative bridge linking molecular, organelle, and cellular scale behaviors.

## MATERIALS AND METHODS

### Materials

All reagents and resources are listed in Table 1.

**Table 1.** Key Reagents and Resources. FBS, fetal bovine serum; MEM, minimum essential medium; BFA, brefeldin A; ATCC, American Type Culture Collection; NIH, National Institutes of Health; NA, not applicable.

Reagent or resource	Source	Identifier
Antibodies		
Anti–α-tubulin monoclonal	Sigma-Aldrich	T5168
Anti–γ-tubulin polyclonal	Invitrogen	PA5-34815
Anti-GM130 (EP892Y) (1 to 100 amino acids)	Abcam	ab52649
Chemicals, peptides, and recombinant proteins		
Thymidine	Sigma-Aldrich	T1895-5G
FBS	MilliporeSigma	F4135
MEM	Life Technologies	11095098
TrypLE Express	Invitrogen	12604021
Puromycin	Gibco	A1113803
RO-3306	Sigma-Aldrich	SML0569
*S*-trityl-L-cysteine	Tocris	2191
Cal-101	Cayman Chemical	15279
GSK-A1	Sigma-Aldrich	SML2453
BFA	InvivoGen	10155-45-01
H89	Abcam	AB120341
8% Glutaraldehyde, EM grade	Electron Microscopy Sciences	16019
16% Paraformaldehyde, EM grade	Electron Microscopy Sciences	15710
Hoechst 33342	Tocris	5117
Experimental models: cell lines		
RBL-2H3 cell	ATCC	Catalog no. CRL-2256; RRID:CVCL_0591
		Wu Lab, cell line ID: B-ae
RBL-2H3 cell stably expressing Cdc42 BD-GFP	First cited in (*39*)	Wu Lab, cell line ID: G-ci
RBL-2H3 cell stably expressing Tks4-GFP	This work	Wu Lab, cell line ID: I-ab
RBL-2H3 cell stably expressing Tks4-GFP and mCherry-FBP17 (L)	This work	Wu Lab, cell line ID: M-aa
Recombinant DNA		
Cdc42 BD-GFP	First cited in (*30*)	Wu Lab, plasmid ID: G011
FBP17-EGFP	First cited in (*39*)	Wu Lab, plasmid ID: F011
FBP17-mCherry	First cited in (*34*)	Wu Lab, plasmid ID: F011d
mCherry-FBP17 (L)	First cited in (*30*)	Wu Lab, plasmid ID: F12
Tks4-GFP	First cited in (*58*)	Wu Lab, plasmid ID: K01
Tks4-mScarlet	This work	Wu Lab, plasmid ID: K01f
Tks5-GFP	Gift from B. Diaz, Sanford-Burnham Medical Research Institute	Wu Lab, plasmid ID: K02
Tks5-mScarlet	This work	Wu Lab, plasmid ID: K02j
GFP-PH_Grp_-PH_Grp_	This work	Wu Lab, plasmid ID: M35a
RFP-PH_TAPP1_	Gift from P. De Camilli	Wu Lab, plasmid ID: M51
GFP-INPP4B	Gift from C. A. Mitchell (*111*)	Wu Lab, plasmid ID: P86
iRFP PH_TAPP1_	This work	Wu Lab, plasmid ID: M52b
RFP-PH_Grp_	Gift from P. De Camilli	Wu Lab, plasmid ID: M35
GFP-PTEN	Addgene	Catalog no. 13039
		Wu Lab, plasmid ID: P91
iRFP	Addgene	Catalog no. 31857
		Wu Lab, plasmid ID: 29
mTagRFP-H2B	Addgene	Catalog no. 58091
		Wu Lab, plasmid ID: H02a
INPP4B Rat shRNA in retroviral untagged vector	OriGene Technologies	TR712257CAGTGGTCGGCAC CATAGAAGTCAGCCTCGTR 712257DAGCCACCTTCTC CTAAGGTCAGCACAGAG
		Wu Lab, shRNA IDs: SRN03 and SRN04
Software and algorithms		
Custom codes for analysis	Written in-house	
Fiji (ImageJ)	NIH	http://fiji.sc/
Prism 10	GraphPad	NA
MATLAB	MathWorks	NA
Python	Python software foundation	NA
Other		
Neon transfection system, 10-μl kit	Life Technologies	MPK1096
35-mm dish | no. 1.5 coverslip | 20-mm glass diameter | uncoated	MatTek Corporation	P35G-1.5-20-C

### Cell culture

RBL-2H3 cells (female, American Type Culture Collection) were cultured in minimum essential medium with Earle’s salts (Invitrogen, 1109580) supplemented with 20% fetal bovine serum (Sigma-Aldrich, F4135) (*39*, *59*, *110*). Cell culture was performed in either a T25 (Corning, 430439) or T75 (Falcon, 353136) cell culture flasks with vented caps. The cells were maintained in a humidified 5% CO_2_ incubator at 37C. The maximum number of cell passages for cells used in our experiments was limited to 30.

### Molecular cloning and plasmids

Cdc42 BD-GFP (Wu Lab, plasmid ID: G011), a biosensor for detecting endogenous active Cdc42 was generated by cloning bacterial expressing Cdc42 activity sensor pET23-CBD-(-PP)-EGFP into pEGFP-N1vector with restriction sites Xho I and Bam HI (*30*). FBP17-EGFP (Wu Lab, plasmid ID: F011) was generated by cloning bacterial expressing EGFP-FBP17 (gift from Pietro De Camilli Lab) into pEGFP-N1 vector with restriction sites Bam HI and Hind III. mCherry-FBP17 (Wu Lab, plasmid ID: F12) was a gift from Pontus Aspenstrom Lab. FBP17-mCherry (Wu Lab, plasmid ID: F011d) was generated by replacing enhanced green fluorescent protein (EGFP) tag in FBP17-EGFP (gift from Pietro De Camilli Lab) to mCherry with restriction sites Xho I and Eco RI. Tks4-GFP (Wu Lab, plasmid ID: K01) was a gift from Begona Diaz Lab. Tks4-mScarlet (Wu Lab, plasmid ID: K01f) is generated by replacing GFP tag in Tks4-GFP with mScarlet3 by Gibson assembly. Tks5-GFP (Wu Lab, plasmid ID: K02) was a gift from Begona Diaz Lab. Tks5-mScarlet (Wu Lab, plasmid ID: K02j) is generated by replacing GFP tag in Tks5-GFP with mScarlet3 by Gibson assembly. GFP-PH_Grp-_PH_Grp_ (Wu Lab, plasmid ID: M35a) was generated by subcloning PH_Grp-_PH_Grp_ into pEGFP vector using restriction site BgI II and Eco RI. RFP-PH_TAPP1_ (Wu Lab, plasmid ID: M51) is a gift from Pietro De Camilli Lab. GFP-INPP4B was a gift from Christina A. Mitchell Lab (*111*). iRFP-PH_TAPP1_ (Wu Lab, plasmid ID: M52b) was generated by subcloning PH_TAPP1_ into iRFP vector using restriction enzyme Kpn I and Hind III. RFP-PH_Grp_ (Wu Lab, plasmid ID: M35) is a gift from Pietro De Camilli Lab. iRFP (Wu Lab, plasmid ID: 29) was purchased from Addgene, catalog no. 5809. GFP-GM130(NLS-AA)-FM (Wu Lab, plasmid ID: O51) was a gift from Jim Rothman Lab. We used a mutated version of GM130 named GM130 (NLS-AA)-FM that does not bind to nucleus as we found that the WT version of GM130 is also strongly associated to nucleus. mTagRFP-H2B (Wu Lab, plasmid ID: H02a) was purchased from Addgene, catalog no. 58091.

### Cell transfection and KD

For transient transection with plasmids, electroporation with the Neon transfection system (Invitrogen, MPK5000) using the 10-ml kit (MPK1025) was used (*39*, *59*). RBL-2H3 cells were trypsinized using trypsin-EDTA (0.25%) (Life Technologies, 25200056), resuspended in 2 ml of RBL-2H3 cell culture medium and counted with a hemacytometer (Marienfeld, 0640010). A total of 1 × 10^6^ RBL-2H3 cells were pelleted, resuspended with 10 ml of the R buffer provided by the transfection kit and mixed with 1 mg of each plasmid used for cotransfection. The electroporation parameters were set as follows: pulse voltage, 1200; pulse width, 20 ms; and pulse number, 2. Following transfection, cells were seeded on a 35-mm glass bottom dishes (MatTek, P35G-1.5-20-C) and incubated overnight.

For KD experiments targeting INPP4B, 1 μg of each respective shRNA was used for each transfection. Four independent shRNAs targeting INPP4B were tested and used in combination. INPP4B KD cells were cultured for 24 hours and then treated with puromycin (1 μg/ml; Gibco, A1113803) for an additional 48 hours before proceeding with imaging experiments.

Stable cell lines expressing Cdc42 BD-GFP or Tks4-GFP were generated by G418 selection of transiently transfected cells and selected with fluorescence-activated cell sorting (FACS). To generate stable cell lines expressing both Cdc42 BD-GFP and mTagRFP-H2B, 1 mg of plasmid of mTagRFP-H2B was transfected into a cell line stably expressing Cdc42 BD-GFP and sorted by FACS 48 hours after transfection.

### Cell synchronization

To investigate how cortical wave dynamics differed as cells transition from interphase to mitosis, we synchronized cells by incubating cells in thymidine, a DNA synthesis inhibitor, for 18 hours arresting a majority of the cells at G_1_-S boundary. Final concentration of 25 mM thymidine was used. After 18 hours of thymidine block, cells were released by 3× phosphate-buffered saline (PBS) washout before addition of fresh medium and imaged 6 to 10 hours later. To synchronize cells after transient transfection, thymidine was added 24 hours after transfection. This protocol allows us to enrich the population of cells that will enter mitosis from G_2_ phase. Synchronization was only used to capture traveling wave conversion from traveling waves from G_2_ to mitosis (Figs. 1A and 4, A to H) and enrichment of mitotic cells for electron microscopy analysis (Fig. 5H). For most of the mitotic waves as well as the scaling curves in Figs. 1E, 3F, and 4 (G and H), unsynchronized cells were imaged by locating cells already in metaphase to minimize synchronized-induced compounding factors.

### Identification of cell cycle stages

For cells with changing wave period in Fig. 4 (C to E), phases were defined by differential interference contrast (DIC) images of chromosomes and by H2B-RFP expression. G_2_ was defined has the phase before Nuclear Envelope Breakdown (NEBD). Prometaphase was defined as the phase after NEBD to before chromosomes are aligned at the metaphase phase, whereas metaphase is the phase from chromosome alignment to before chromosomes separate and furrow initiation. For each phase, the time length used for fast Fourier transform (FFT) analysis was 10 min.

### TIRF imaging

TIRF experiments were performed using a Nikon TiE inverted microscope equipped with three laser lines (488, 561, and 642 nm) (*110*). The microscope was fitted with an iLas2 motorized TIRF illuminator (Roper Scientific) and with a Prime 95B sCMOS camera (Photometrics). Nikon objectives including Apo TIRF 1003 [numerical aperture (NA), 1.49, oil] and 603 (NA, 1.49, oil) were used for image acquisition. Sequential excitation with lasers at 491-, 561, or 642 nm was used for imaging cells in two or three channels. A quad–band-pass filter (Di01-R405/488/561/635, Semrock) and single-band filters including 520/35 nm for GFP, 609/54 nm for RFP and mCherry, and 692/40 nm for iRFP (Semrock) were used accordingly. The microscope operation was controlled by MetaMorph software (Universal Imaging). Throughout the experiments, imaging was performed at a constant temperature of 37C using an on-stage incubator system (Live Cell Instrument, Seoul, South Korea). Humidified CO_2_ (5%) was supplied for all live imaging. Most live-cell images were taken in 1- to 4-s intervals. For long-term imaging in Fig. 3 (G to I), multiple stage positions were used for each experiment to increase the number of cells imaged. Images were acquired with 2-min intervals. The acquired images were in 16-bit format and had dimensions of 1200 × 1200 pixels. The pixel size was 0.183 μm/pixel when imaged with a 60× objective and 0.11 μm/pixel when imaged with a 100× objective.

### Drug treatment

For inhibitor, experiments, GSK-A1, Cal-101, H89, BFA, and Golgicide A were further diluted from frozen stocks with prewarmed medium to 2× final concentration. The inhibitors were added during imaging to achieve the desired final concentrations. For wave perturbation experiments, a final concentration of 5 nM GSK-A1, 88 nM Cal-101, 10 to 20 μM Golgicide A, 0.2 to 3.5 μM BFA, or 50 to 100 μM H89 was added during imaging. For Cdk1 inhibitor RO-3306 treatment, cells were synchronized using thymidine for 18 hours and released for 5 hours before imaging. Acute treatment of RO-3306 (2 to 10 μM) was added during imaging.

### Wave analysis

#### Color-coded time-projection images for wave propagation visualization

To visualize spatiotemporal wave propagation (Fig. 1A), we developed a MATLAB code that generates composite color images from time-lapse microscopy data. The method encodes temporal progression into pseudocolor. Each frame was intensity normalized by contrast adjustment to enhance dynamic range while minimizing saturation artifacts. For each selected time interval, three time frames were extracted at fixed temporal offsets. These frames were then mapped to the red, green, and blue color channels, respectively.

#### Kymograph, montages, and intensity profile analysis

For the generation of kymographs, Fiji software was used. The “reslice” tool and “average projection filter” were applied. Five to 10 slices were used to generate the average projection. Sequential montages were generated using the “Make Montage” tool. For intensity profiles, data pointed were obtained using the average intensity profile of a region of interest (ROI) of 20 × 20 pixels.

#### Fourier transform

Oscillation was determined by visual impression of kymographs and confirmed using the built-in FFT function (described below) in MATLAB. Cells that gave an output of a single FFT peak within the designated range (typically 10 to 100 s) were considered positive for oscillations, and cells without the FFT peak were considered negative for oscillations. To identify major periodic components and visualize power spectra of intensity profile, a custom MATLAB FFT code is used. The code extracts temporal features from an input image intensity profile by preprocessing, filtering, and computing the FFT. The input profile was obtained using the average intensity profile of a ROI of 20 × 20 pixels. It is first converted to double precision and detrended using third-degree polynomial fitting to remove low-frequency drift, followed by application of a Hanning window to minimize spectral leakage and normalization to the range based on the profile’s minimum and maximum values. Next, the FFT is performed with zero padding to the nearest power of 2, and the resulting frequency-domain data are used to calculate the power spectrum as the squared magnitude of the FFT coefficients. Low-frequency artifacts are excluded by selecting components above a cutoff (0.025 normalized frequency), and the power is plotted against both frequency and period to reveal oscillatory dynamics. Peaks in the power spectrum are detected by comparing neighboring values within a radius, highlighting significant periodic signals. For each major peak, the period, power, and percentage contribution relative to the filtered spectral area are computed, annotated on the plots, and saved for quantitative analysis and figure export.

#### Rise and fall phases

An automated MATLAB-based algorithm was used to quantify dynamic features in signal intensity traces. Normalized traces were analyzed using the findpeaks function, with user-defined thresholds for minimum peak distance and minimum peak height to identify local maxima. To detect valleys, the first derivative of each trace was computed using the gradient function, and valley points were defined as local minima where the derivative changed from negative to positive. For each identified peak, the algorithm determined the nearest preceding and following valley points. The rise duration was calculated as the number of frames between the preceding valley and the peak, while the drop duration corresponded to the interval between the peak and the subsequent valley. Additionally, the time to the next peak was measured. Scatterplot of two extracted parameters (rise and drop durations versus time to the next peak) was generated for further statistical analysis. For visual verification, plots were generated displaying the original trace with peaks marked in red and valleys in blue (fig. S2E).

#### Wavelet analysis

To quantify the temporal evolution of wave periods, we performed a continuous wavelet transform (CWT) on intensity traces extracted from ROIs. Briefly, fluorescence time series were first mapped to an experimental time vector based on the imaging interval. The CWT was computed using MATLAB’s built-in CWT function with a Morlet wavelet, returning complex coefficients across time and scale. Scales were converted to oscillation periods (minutes). The output was plotted in two panels: (i) the raw intensity trace over time and (ii) a scalogram showing wavelet power as a function of both time and period. To highlight oscillatory modes across a broad dynamic range, wavelet power was log-transformed before visualization. Color axes were scaled to emphasize weaker oscillatory signals, and results were plotted using the jet color map. The MATLAB codes used to generate figures have been previously documented and are accessible on GitHub (https://github.com/min-wu-lab, including https://github.com/min-wu-lab/2023-Tong-et-al and https://github.com/min-wu-lab/2026-Fung-et-al).

### Bipolar spindle sample preparation, spinning disk confocal imaging, and quantification

Cells are seeded on 35-mm MatTek dishes at a plating density of 20,000 cells/mm^2^ for 24 hours before chemical fixation using 4% paraformaldehyde (Electron Microscopy Sciences) for 15 min. Cells are washed with 1× PBS (Gibco), permeabilized with 0.3% NP-40 (Sigma-Aldrich) and 0.05% Triton X-100 (TX-100; Sigma-Aldrich) in PBS, and incubated with primary antibodies at 4° overnight. Cells were washed with 0.05% NP-40 and 0.05% TX-100 diluted in PBS. Secondary antibody incubation is 1 hour at room temperature. Cells were stained with Hoechst 33342 before imaging.

Spinning-disk confocal imaging was performed using Andor Dragonfly 200 (Oxford Instruments) inverted microscope with a Zyla cMOS 5.5 camera and controlled by Fusion (Oxford Instruments) software. DAPI (405 nm), GFP (488 nm), and RFP (561 nm) lasers were used. Images were acquired with a PlanApo objective (60×, 1.49 NA). 3D stacks at *z*-spacing of 0.2 mm are acquired to ensure the capture of entire cell.

We define spindle length as the distance between two centrosomes indicated by immunostaining of anti–γ-tubulin, dilution of 1:1000 (Invitrogen, PA5-34815) in mitotic cells. In addition, cells are stained with anti–α-tubulin, dilution of 1:1000 (Sigma-Aldrich, T5168), to ensure that the fluorescently labeled spindles correlate with spindle pole distance. 3D segmentation considers spindle pole positions that are not on the same *z* plane. Cells stained with anti–α-tubulin antibody show cytoplasmic fluorescence, which we used to measure cell dimensions. Spindle width was quantified from maximum-intensity projections of confocal *z*-stacks by measuring the width of α-tubulin signal at the spindle equator. All images for spindle width analysis were acquired and processed identically and measurements were restricted to spindles oriented approximately parallel to the imaging plane. Spindle width values represent apparent two-dimensional widths in the imaging plane and were used for relative comparisons between experimental conditions. Spindle volume was quantified using the Spindle3D plugin (*112*) in two-color confocal image stacks costained for α-tubulin and Hoechst 33342.

### Monopolar spindle sample preparation, super-resolution imaging, and quantification

Cells were synchronized with thymidine for 18 hours before releasing into normal medium. After 5 hours, cells were treated with STLC (2.5 μM) for 2 hours before fixation. Cells are preextracted with 0.2% saponin for 45 min and fixed with 3% paraformaldehyde (Electron Microscopy Sciences) and 0.1% glutaraldehyde (Electron Microscopy Sciences) for 15 min. After three washes in PBS, cells were permeabilized and blocked with 3% BSA (Jackson ImmunoResearch) and 0.2% TX-100 (Sigma-Aldrich). Cells were then incubated with primary antibodies in 3% BSA (Jackson ImmunoResearch) and 0.2% TX-100 (Sigma-Aldrich) at 4°C overnight. After three washes (three times for 5 min), cells were incubated with secondary antibodies for 1 hour at room temperature. After three washes (three times for 5 min), the cells were postfixed with 3% paraformaldehyde (Electron Microscopy Sciences) and 0.1% glutaraldehyde (Electron Microscopy Sciences) for 10 min. Cells were washed three times with 1× PBS (Gibco).

Monopolar spindles were imaged by DNA-PAINT imaging. Buffer composition (PBS, 500 mM NaCl, 20 mM Na_2_SO_3_, and 1 mM Trolox) and fluorogenic imager probes (Cy3B-BHQ2) were previously reported (*113*). Before imaging, the cells were rinsed three times with freshly made DNA-PAINT imaging buffer. Beads (100-nm diameter; Thermo Fisher Scientific, F8801) were then added to the sample at a dilution of 1:1,000,000 to calibrate experimental point spread function (PSF). Last, the imager probes were added to the sample at a final concentration of 5 nM. Samples were imaged on a custom-built microscope based on Olympus IX81 inverted stand with a 100× 1.35-numerical aperture silicone oil-immersion objective and an sCMOS camera (Hamamatsu, ORCA-Flash 4.0 V3). For illumination, a 560-nm laser (MPB Communications) and an acousto-optical tunable filter (AA Optics) for intensity modulation were used with a dichroic (Chroma, ZT405/488/561/640). A cylindrical lens (Thorlabs, *f* = 1000 mm) was added to the emission path to introduce astigmatism for 3D imaging (*114*). Two filters (Semrock, BLP02-561R-25; and Semrock, BrightLine 609/54) were placed before the camera for fluorescence detection. An orientation-independent DIC (OI-DIC) channel was integrated into this microscope for transmitted-light imaging as needed. A software-based drift tracking with a feedback loop was implemented to stabilize the focal plane. When the drift tracking was activated, the live-view transmitted-light image was cross-correlated with a precalibrated *z*-stack to determine the axial drift. The focal plane was then adjusted accordingly. Image data acquisition and analysis were conducted with a custom-programmed open-source software package PYthon Microscopy Environment (PYME).

For image acquisition, an astigmatic experimental PSF for DNA-PAINT was calibrated by taking a *z*-stack of 100-nm-diameter beads in the sample with 50-nm step size and 150 slices. After that, a field of view was selected and a *z*-stack of six raw DIC images with two orthogonal shear directions and three biases crossing the cell center were taken. This was followed by acquiring background reference images using the same parameter settings for OI-DIC reconstruction. Next, a *z*-stack with a 200-nm step size was taken in the transmitted-light channel for calibrating the software-based drift tracking, which was activated afterward. DNA-PAINT data were then recorded at 100 Hz with a laser intensity of ~13 kW cm^−2^ delivered to the sample. *Z*-stacking was performed with a 300-nm step size to cover the spindle in 3D. Frames (~25,000) were recorded at each focal plane.

DNA-PAINT data were analyzed and visualized with PYME (*115*) and custom code written in Python. Localizations were performed in 3D by fitting with the acquired experimental PSF (*116*). Localization positions were then corrected for lateral drift according to the recorded drift from the software-based drift tracking and by using a cross-correlation algorithm (*117*). To quantify α-tubulin radial density profiles from the drift-corrected DNA-PAINT images, concentric annuli centered on the spindle pole were defined, and 3D localizations from a defined *z*-thickness (4 μm) were assessed by cylindrical panorama projections at selected radii from 1 to 6 μm, each ring with a thickness of 200 nm. The α-tubulin density plots are generated at radii from 0 to 10 μm, with each ring a thickness of 200 nm. The radial density was quantified as the number of localizations within each annulus divided by its area in the panorama projection. The density profile for each cell was then normalized to its maximum value. Although DNA-PAINT targeted α-tubulin, low-level nonspecific bindings were present throughout the cytoplasm, allowing an approximate segmentation of the cell boundary. The cell size was, therefore, estimated by thresholding the DNA-PAINT images and applying the wand tracing tool in Fiji (*118*).

### Electron microscopy

Cells were synchronized and grown on coverslips. Samples were fixed in 2.5% glutaraldehyde in 0.1 M sodium cacodylate buffer (pH 7.4) for 1 hour and then rinsed in the same buffer. Postfixation was carried out on ice for 1 hour in 0.5% osmium tetroxide and 0.8% potassium ferrocyanide in buffer. Samples were rinsed with sodium cacodylate buffer and distilled water and then en bloc stained with 2% aqueous uranyl acetate for 1 hour on ice. After additional rinses in distilled water, cells were dehydrated through a graded ethanol series to 100% ethanol.

Samples were infiltrated with Embed 812 resin (Electron Microscopy Sciences). Gelatin capsules filled with resin were inverted onto the coverslips and polymerized at 60°C for 24 hours. The hardened blocks were separated from the coverslips and sectioned at 60 nm using a Leica UltraCut UC7 ultramicrotome. Sections were collected on formvar/carbon-coated grids and contrast stained with 2% uranyl acetate followed by lead citrate. Grids were imaged using a FEI Tecnai Biotwin transmission electron microscope operating at 80 kV, and images were acquired with an AMT NanoSprint15 MK2 sCMOS camera.

### Statistical analysis

Statistical analyses were performed using GraphPad Prism9. For the scaling plots (Figs. 1E and 3C), data were binned by averaging every five consecutive data points. To compare changes between two groups of data points, unpaired two-tailed Student’s *t* test was used. Data were shown as means ± SD. Please refer to the figures and figure legends for the number of cells used for quantification per condition. *N* refers to number of independent experiments, and *n* refers to number of independent cells. For mitotic waves, typically cells with mitotic waves were first identified visually. For experiments where cells were randomly chosen, *n* = 27 of 30 mitotic cells refer to 27 of the 30 imaged cells showing the representative phenotypes.
