# Supplementary material for: Mitotic Cdc42 waves encode PI(3,4)P2 signaling and Golgi morphological state to control spindle scaling
Source: Sci Adv. 2026 Jun 19;12(25):eaec7705. doi: 10.1126/sciadv.aec7705 (PMC13281794; doi:10.1126/sciadv.aec7705)
Supplement: Supplementary file 1 — Figs. S1 to S7 Legends for movies S1 to S6 [file sciadv.aec7705_sm.pdf]

Science Advances
AAAS

Supplementary Materials for

Mitotic Cdc42 waves encode PI(3,4)P_2 signaling and Golgi morphological state to control spindle scaling

Suet Yin Sarah Fung *et al.*

Corresponding author: Min Wu, wu.min@yale.edu

Sci. Adv. **12**, eaec7705 (2026)
DOI: 10.1126/sciadv.aec7705

The PDF file includes:

Figs. S1 to S7
Legends for movies S1 to S6

Other Supplementary Material for this manuscript includes the following:

Movies S1 to S6

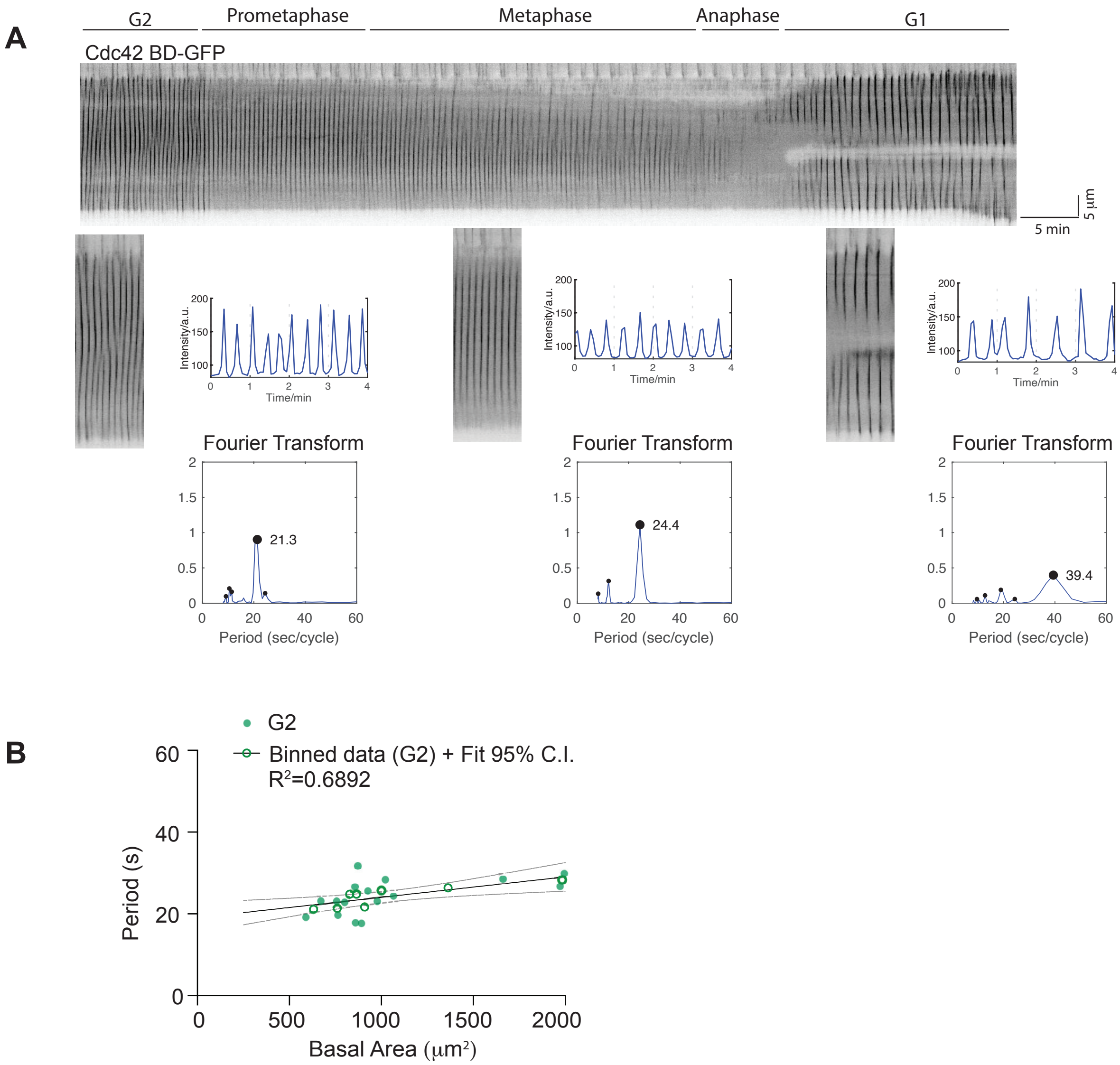

Figure S1. Changes in wave periods during cell cycle progression

(A) Intensity profiles and corresponding Fourier transform analyses from the kymograph of a Cdc42BD–GFP–expressing cell during mitotic progression (Figure 1C).

(B) Scatter plot of wave periods measured in G2 plotted against mitotic cell size (n = 16 cells). Filled circles represent individual cells, and open circles indicate binned data. The solid line shows the linear regression fit, and dashed lines denote the 95% confidence interval (C.I.).

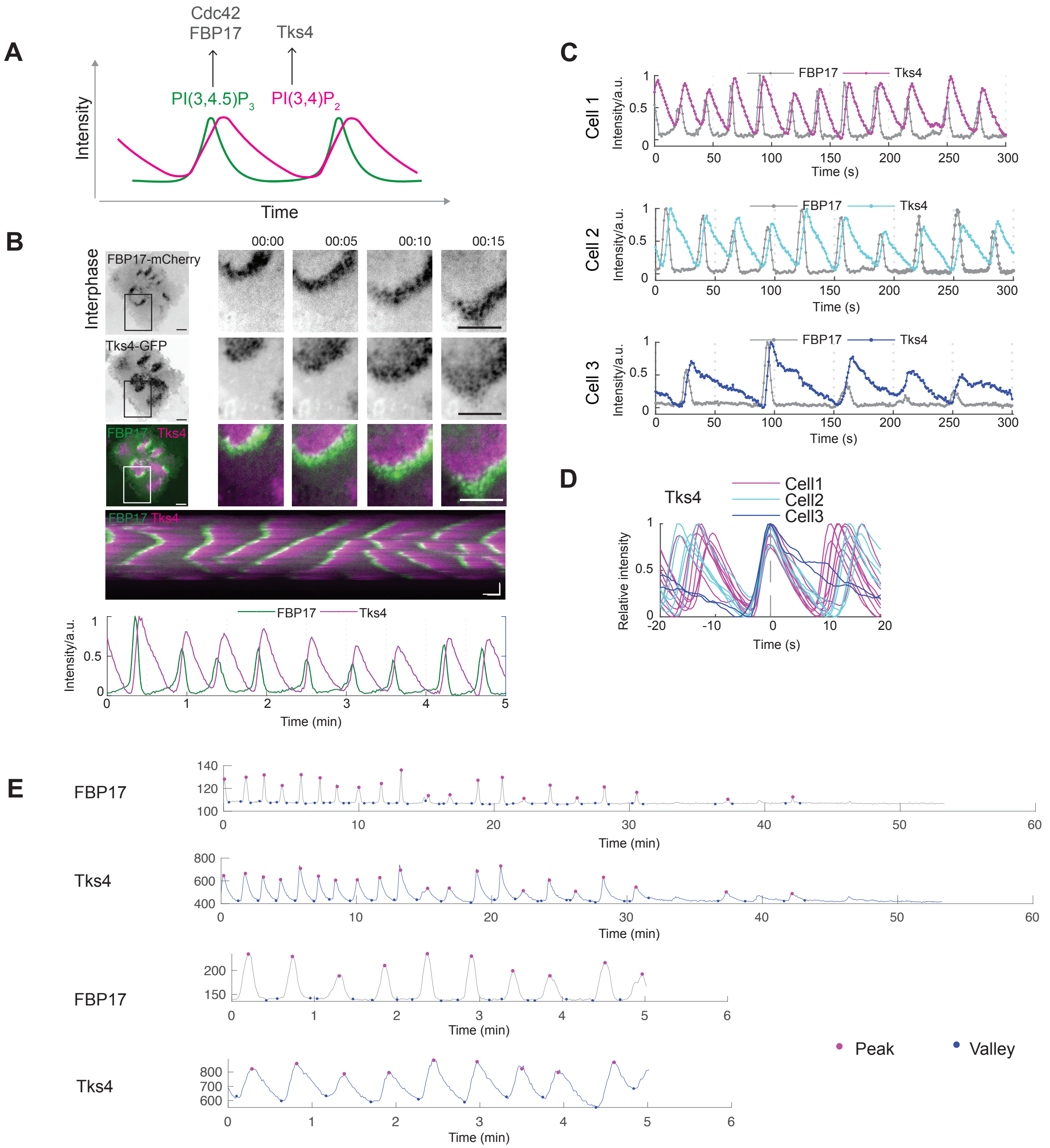

Figure S2.Characterization and quantification of wave periods

(A) Schematic representation of coupled waves of PI(3,4,5)P3 and PI(3,4)P2, as well as their effectors, Cdc42 and FBP17.

(B) Representative TIRF images, sequential montage, kymographs and intensity plots of a region of an interphase cell co-expressing FBP17-mCherry and Tks4-GFP.

(C-D) Intensity profiles (C) and overlay (D) of three representative cells expressing FBP17-mCherry and Tks4-GFP with different wave periods.

(E) Quantification of peaks (pink dots) and valleys (blue dots) for two representation traces of cells expressing FBP17-mCherry and Tks4-GFP respectively.

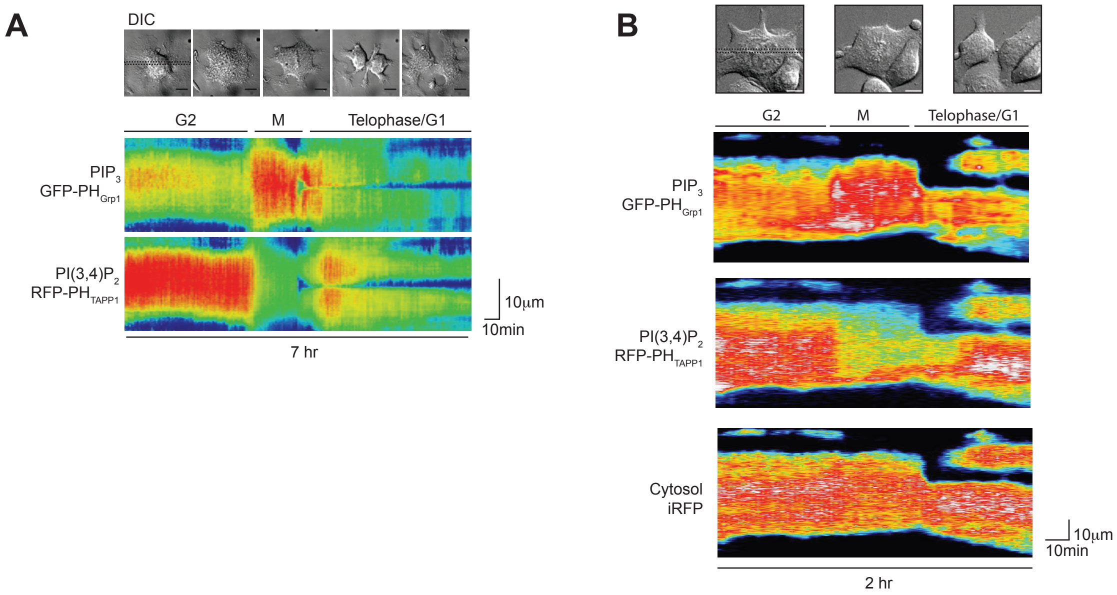

Figure S3. Changes in PIP levels during cell cycle
(A) Representative DIC images corresponding to kymograph from TIRF imaging in Figure 2G.
(B) Representative DIC images and kymographs from TIRF imaging of a cell expressing GFP-PH_{Grp}, sensor of PI(3,4,5)P_3, RFP-PH_{TAPP1}, sensor for PI(3,4)P_2 and iRFP cytosolic control. Scale bar; 10min ((n=3/3 cells, 2 independent experiments).
Dashed boxes indicate regions used to generate kymographs.Images scale bar; 10μm. Kymograph horizontal scale bar; 10min, vertical scale bar; 10μm.

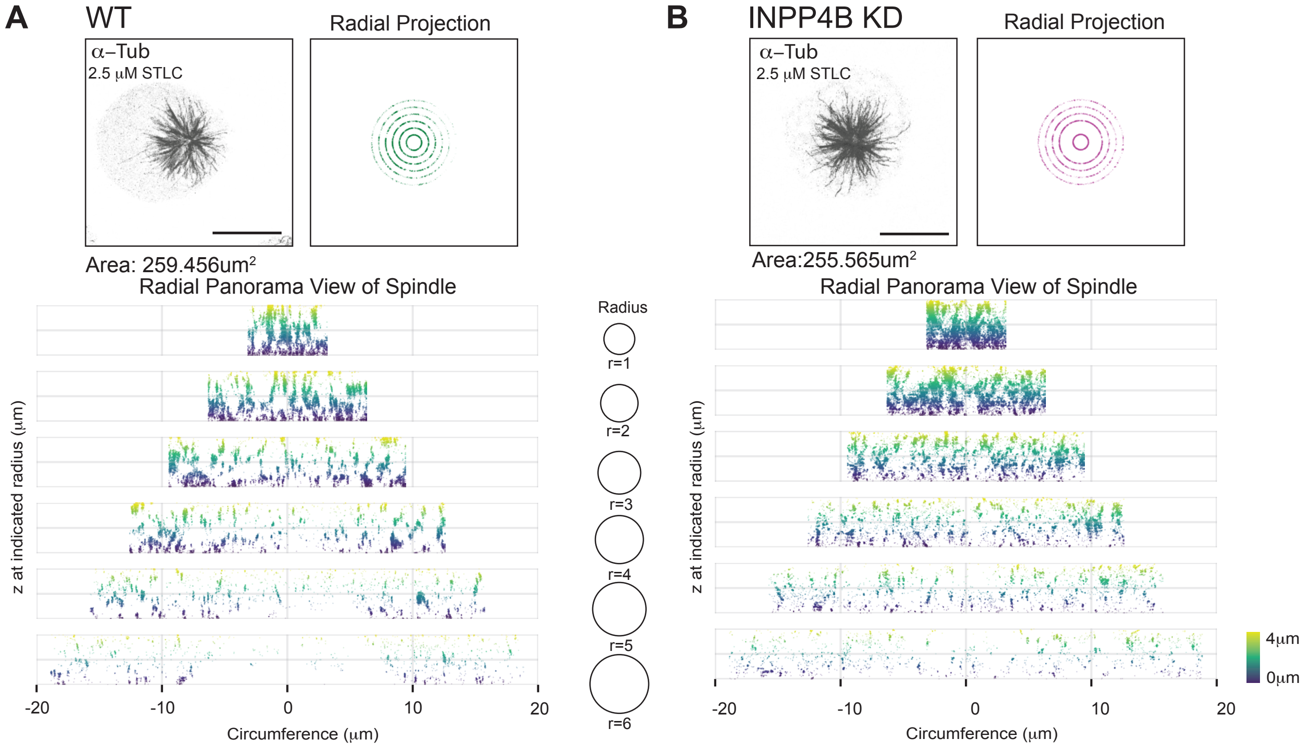

Figure S4. Changes in microtubule density in WT and INPP4B KD characterized by DNA-PAINT imaging of spindles
(A–B) Representative single-molecule DNA-PAINT images of monopolar spindles (top left), with corresponding radial projections of α-tubulin localization density (top right). Radial panorama views at the indicated distances (bottom) illustrate α-tubulin intensity at increasing radii. WT (A) and INPP4B KD (B) cells are presented as size-matched pairs to enable direct comparison of radial density decay profiles.

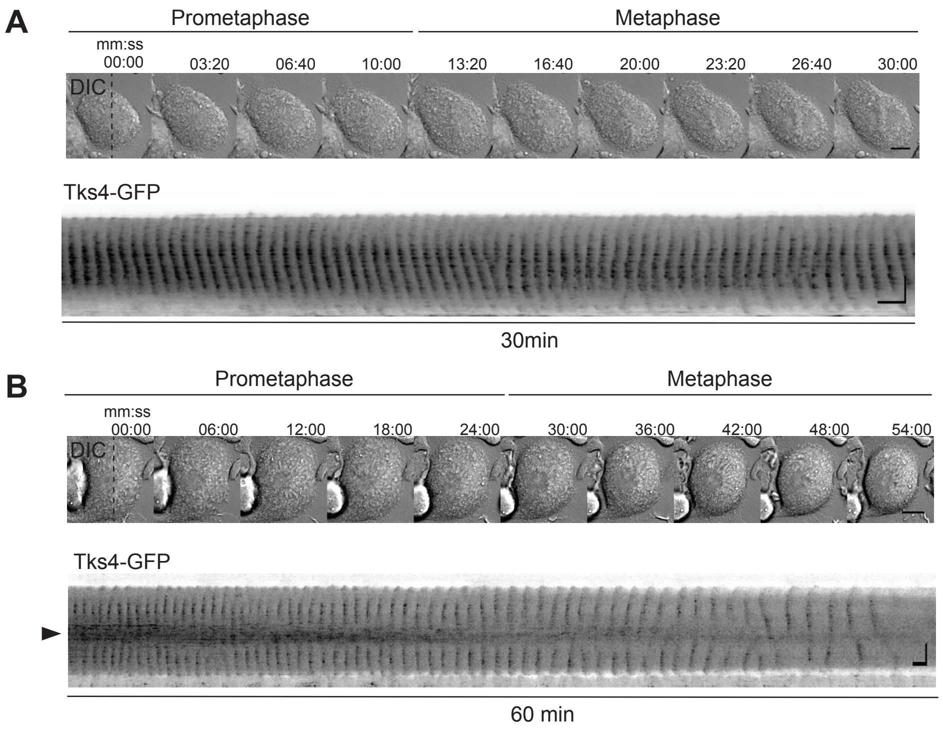

Figure S5. Cluster formation in mitotic cells associated with dynamic changes in wave period
(A-B) Representative montages of DIC images and kymograph of a mitotic cell expressing Tks4-GFP with constant wave period (A) and changing wave period (B). Dashed lines indicate regions used to generate kymographs. Clusters (indicated with arrowhead) are visible only with changing wave periods.
Images scale bar; 10μm. Kymograph horizontal scale bar; 1min, vertical scale bar; 10μm.

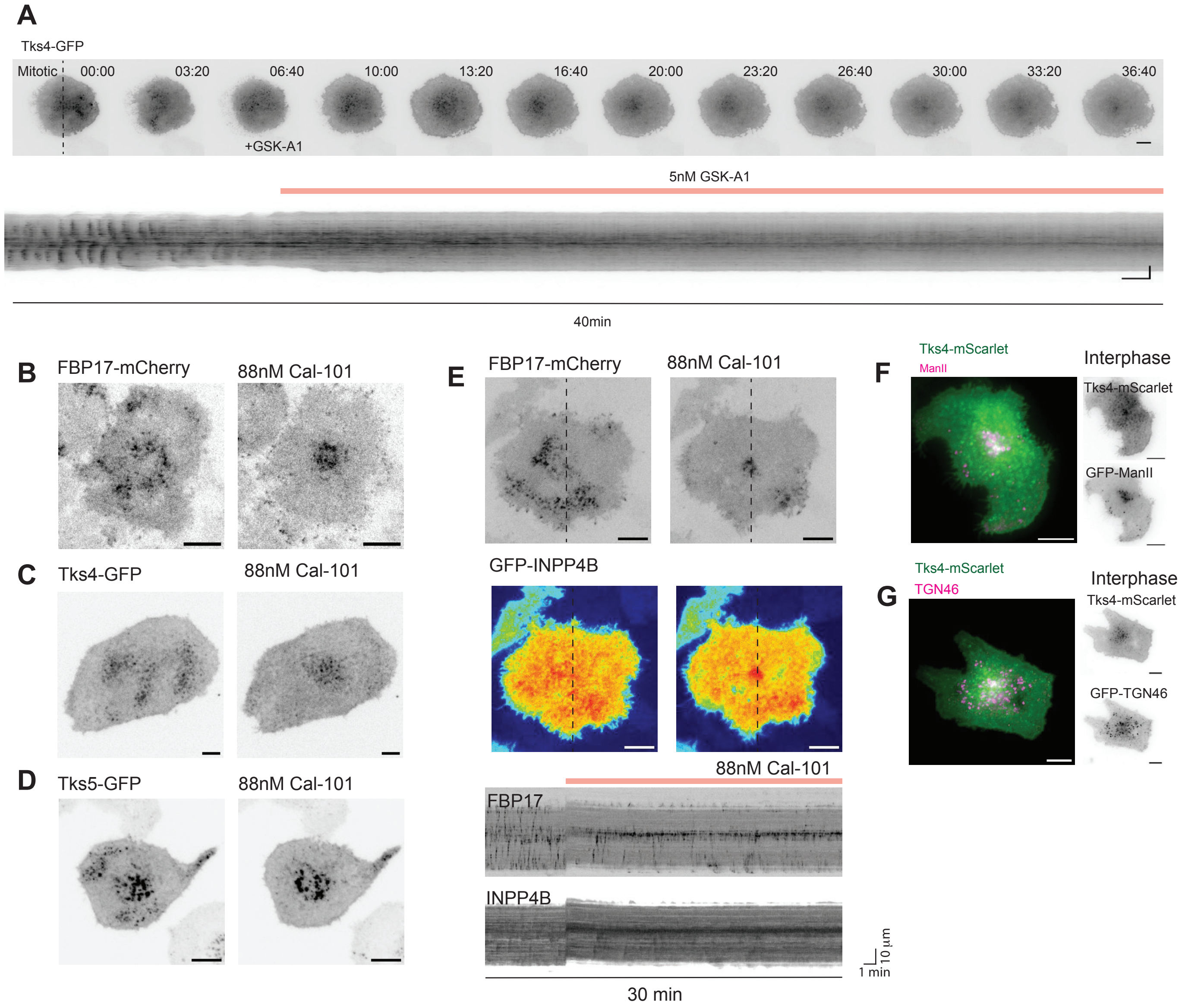

Figure S6. Wave-associated proteins dynamically redistribute between plasma membrane waves and intracellular compartments
(A) Before and after TIRF images and kymograph of a mitotic cell expressing Tks4-GFP treated with 5nM GSK-A1.
(B-D) TIRF images of cells expressing FBP17-mCherry (B), Tks5-GFP (C) and Tks4-GFP (D) before and after 88nM Cal-101 treatment.
(E) TIRF images and kymographs of cells expressing FBP17-mCherry and GFP-INPP4B before and after 88nM Cal-101 treatment
(F-G) Merged TIRF images of cell expressing Tks4-mScarlet with GFP-ManII (F) and GFP-TGN46(G).
Dashed line indicate region used to generate kymographs. Images scale bar; 10μm. Kymograph horizontal scale bar; 1min, vertical scale bar; 10μm.

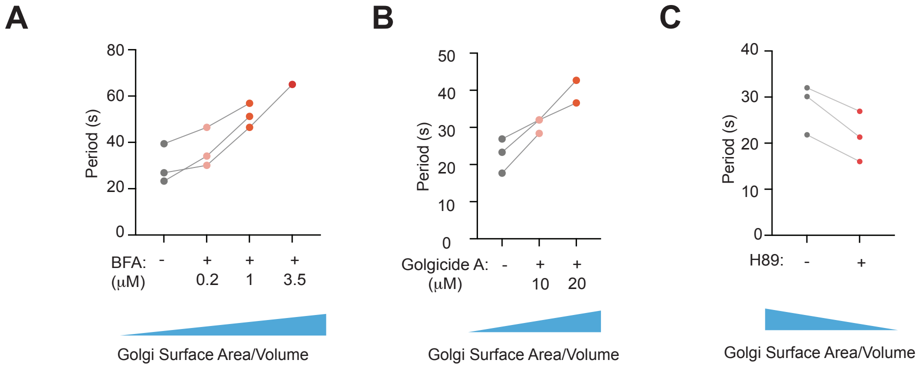

Figure S7. Pertubration of Golgi dynamics tunes mitotic wave periods
Period of Cdc42 waves from representative cells treated with increase doses of BFA (A), Golgicide A (B) and H89 (C).

Supplementary Videos

Supplementary Video 1
Cdc42 waves in RBL-2H3 cell throughout different cell cycle.
TIRF video of a cell expressing Cdc42-BD-GFP (green) with DIC was acquired at 4 sec per frame, play rate is 10fps, 40x real time. Scale bar 10μm.

Supplementary Video 2
TIRF movie and kymograph of Cdc42-BD-GFP waves in mitotic RBL-2H3 cells of different sizes. Blue box indicates region of kymograph. Movies were acquired at 4 sec per frame, play rate is 5fps, 20x real time. Scale bar 10μm.

Supplementary Video 3
Coordinated waves of FBP17(green) and Tks4(magenta) in RBL-2H3 interphase cell.
TIRF movies of cell expressing FBP17-mCherry and Tks4-GFP was acquired at 1 sec per frame, play rate 5 fps, 5x real time. Scale bar 10μm.

Supplementary Video 4
Coordinated waves of FBP17(green) and Tks4(magenta) in RBL-2H3 mitotic cell.
TIRF movies of cell expressing FBP17-mCherry and Tks4-GFP was acquired at 4 sec per frame, play rate 5 fps, 20x real time. Scale bar 10μm.

Supplementary Video 5
TIRF movie and kymograph of Tks4 waves in mitotic RBL-2H3 cell.
Mitotic cell expressing Tks4-GFP was was acquired at 4 sec per frame, play rate 20fps, 80x real time. Scale bar 10μm.

Supplementary Video 6
TIRF movie of FBP17 waves in RBL-2H3 cell after GSK-A1 (1nM-10nM) treatment. Scale bar 10μm.
